# Supplementary material for: Mixed model-based deconvolution of cell-state abundances (MeDuSA) along a one-dimensional trajectory
Source: Nat Comput Sci. 2023 Jul 13;3(7):630–43. doi: 10.1038/s43588-023-00487-2 (PMC10766563; doi:10.1038/s43588-023-00487-2)
Supplement: Supplementary file 1 — Supplementary Figs. 1–31, Note, and Tables 1 and 2. [file 43588_2023_487_MOESM1_ESM.pdf]

nature computational science

Article https://doi.org/10.1038/s43588-023-00487-2

Mixed model-based deconvolution of cell-state abundances (MeDuSA) along a one-dimensional trajectory

In the format provided by the authors and unedited

Supplementary Note (sections 1-6)

Supplementary Figures 1-31

Supplementary Tables 1-2

Supplementary References

Supplementary Note

Section 1. Selecting signature genes

We employ the generalized additive model (GAM) to associate cellular gene expression levels with a pre-defined cell-state trajectory. For a gene across cells, the GAM can be written as:

$$\mathbf{Y} = s(\mathbf{T}) + \mathbf{U}\boldsymbol{\alpha} + \mathbf{e} \qquad (\mathbf{1})$$

where $\mathbf{Y}$ is an $n \times 1$ vector of gene expression levels across n cells; $\mathbf{T}$ is an $n \times 1$ vector of the states of the cells with $s(\mathbf{T})$ being the smoothing spline; $\mathbf{U}$ is an incidence matrix of the covariates (e.g., donor and sequencing depth) with $\boldsymbol{\alpha}$ being the corresponding effects; $\mathbf{e}$ is an $n \times 1$ vector of residuals, $\mathbf{e} \sim N(0, \mathbf{I}\sigma_e^2)$. Note that the GAM described here assumes a normal distribution to the residuals but can be extended to consider other distributions, such as a negative binomial distribution[1,2].

The smoothing spline $s(\mathbf{T})$ is modeled as a linear combination of k cubic basis functions:

$$s(\mathbf{T}) = \sum_{i=1}^{k} b_i(\mathbf{T})\beta_i \qquad (2)$$

where b_i denotes the i cubic basis function with β_i being the corresponding effect. The cubic smoothing spline $s(\mathbf{T})$ is a piecewise cubic polynomial whose first and second derivatives are continuous at each knot for converting the cell states ($\mathbf{T}$) into the non-linear space. These transformed cell states are then associated with the cellular gene expression levels by a multivariable linear regression.

The number of knots plays a key role in model fitting. A too large (small) number could lead to overfitting (underfitting) of the GAM. To balance the fidelity and smoothness of the model, we can employ a roughness penalty:

$$\mathrm{RSS}(s, \lambda) = \sum_{\mathrm{i}=1}^{p} \left(Y_i - s(Y_i)\right)^2 + \lambda \int [s''(x)]^2 dx \qquad (3)$$

where s_g'' is the second derivates of the smoothing spline with λ being the shrinkage parameter. In matrix notation, supplementary Eq. (3) can be written as

$$\mathrm{RSS}(s, \lambda) = (\mathbf{Y} - \mathbf{b}\boldsymbol{\beta})'(\mathbf{Y} - \mathbf{b}\boldsymbol{\beta}) + \lambda\boldsymbol{\beta}'\boldsymbol{\Omega}\boldsymbol{\beta} \qquad (\mathbf{4})$$

with $\{\boldsymbol{\Omega}\}_{ij} = \int s_i''(x)s_j''(x)\,dx$. $\boldsymbol{\beta}$ can be estimated using the least squares approach:

$$\boldsymbol{\beta} = (\mathbf{b}'\mathbf{b} + \lambda\boldsymbol{\Omega})^{-1}\mathbf{b}'\mathbf{Y} \qquad (5)$$

The shrinkage parameter λ is unknown but can be determined by the generalized cross-

validation method[3]. With the roughness penalty, we can place enough knots (20, by default) to ensure the fidelity of the GAM while control the curvature of the fitted curve by shrinking the regression coefficients (Supplementary Figure 30).

We use the Wald chi-squared statistic below to test against the null hypothesis that $\mathrm{H}_0: \mathbf{C}'\boldsymbol{\beta} = 0$ (i.e., the expression level of the gene is not associated with the cell-state trajectory):

$$W = \boldsymbol{\beta}'(\mathbf{C}'\boldsymbol{\Sigma}\mathbf{C})^{-1}\mathbf{C}'\boldsymbol{\beta} \tag{6}$$

where $\boldsymbol{\beta}$ is a $k \times 1$ vector of the regression coefficient of each cubic basis function with K being the number of knots; $\boldsymbol{\Sigma}$ is a $k \times k$ variance-covariance matrix of $\boldsymbol{\beta}$; $\mathbf{C}$ is a $k \times c$ matrix of the contrasts. We rank genes according to their Wald test chi-squared values. That is, genes with higher chi-squared values are regarded as more informative genes to distinguish cells among different cell states. To avoid over-representation of some cell states, we divide the cell-state trajectory into d intervals ($d = 10$, by default) and assign each gene to a specific interval in which it attains the highest mean expression. For each interval, certain top informative genes are selected as signature genes.

Section 2. Details of the derivation and parameter estimation for Equation 3 in the Main Text

According to the Brooks lemma[4,5], if we define the sample space Ω as the set of all possible realizations of $\boldsymbol{\pi} = (\pi_1, \pi_2, \dots, \pi_n)$, i.e., $\Omega = \{\boldsymbol{\pi}: P(\boldsymbol{\pi}) > 0\}$, then for any two realizations $\boldsymbol{\mu}$ and $\boldsymbol{\nu} \in \Omega$, we have

$$\frac{P(\boldsymbol{\mu})}{P(\boldsymbol{\nu})} = \prod_{i=1}^{n} \frac{P(\mu_i|\mu_1, \dots, \mu_{i-1},\ \nu_{i+1}, \dots, \nu_n)}{P(\nu_i|\mu_1, \dots, \mu_{i-1},\ \nu_{i+1}, \dots, \nu_n)}$$

It is described in main-text Eq. (2) that the abundance of cell i can be modeled as

$$\alpha_i = \theta \sum_{j=1, j\neq i}^{k} w_{ij}\alpha_j + \epsilon_i \tag{7}$$

where the definitions of the parameters are the same as those in the main text. The marginal distribution of α_i is $\alpha_i \sim N(\theta \sum_{j=1, j\neq i}^{k} w_{ij}\alpha_j, \sigma_{\epsilon(i)}^2)$.

Let $\boldsymbol{\mu}$ be a $k \times 1$ vector of random variables with $\boldsymbol{\mu} = \boldsymbol{\alpha} = (\alpha_1, \alpha_2, \dots, \alpha_k)$ and $\boldsymbol{\nu}$ be a $k \times 1$ vector of zeros, i.e., $\boldsymbol{\nu} = \mathbf{0}$. We then have

$$\frac{P(\boldsymbol{\mu})}{P(\mathbf{v})} = \prod_{i=1}^{k} \frac{\exp\{-\frac{1}{2\sigma_{\epsilon(i)}^2}(\alpha_i - \theta \sum_{j<i} w_{ij}\alpha_j - \theta \sum_{j>i} w_{ij}0)^2\}}{\exp\{-\frac{1}{2\sigma_{\epsilon(i)}^2}(0 - \theta \sum_{j<i} w_{ij}\alpha_j - \theta \sum_{j>i} w_{ij}0)^2\}}$$

where ϵ_i and ϵ_j are assumed to be independent and identically distributed. Since $w_{ij} = w_{ji}$, $w_{ii} = 0$, and $P(\mathbf{v}) = 1$, we have

$$P(\mathbf{x}) \propto \prod_{i=1}^{k} \exp\{-\frac{1}{2\sigma_\epsilon^2}(\alpha_i{}^2 - 2\alpha_i\theta \sum_{j<i} w_{ij}\alpha_j)\}$$

$$\propto \prod_{i=1}^{k} \exp\{-\frac{1}{2\sigma_\epsilon^2}(\alpha_i{}^2 - \theta \sum_{j=1}^{k} \alpha_i w_{ij}\alpha_j)\}$$

$$\propto \exp\{-\frac{1}{2\sigma_\epsilon^2}(\sum_{i=1}^{k}(\alpha_i - 0)^2 - \theta \sum_{i=1}^{k}\sum_{j=1}^{k}(\alpha_i - 0)w_{ij}(\alpha_j - 0))\}$$

$$\propto \exp\{-\frac{1}{2\sigma_\epsilon^2}((\boldsymbol{\alpha} - \mathbf{0})^{\mathrm{T}}\mathbf{I}(\boldsymbol{\alpha} - \mathbf{0}) - (\boldsymbol{\alpha} - \mathbf{0})^{\mathrm{T}}\theta\mathbf{W}(\boldsymbol{\alpha} - \mathbf{0}))\}$$

$$\propto \exp\{-\frac{1}{2}(\boldsymbol{\alpha} - \mathbf{0})^{\mathrm{T}}((\mathbf{I} - \theta\mathbf{W})^{-1}\sigma_\epsilon^2)^{-1}(\boldsymbol{\alpha} - \mathbf{0})\}$$

where $\mathbf{W} = \{w_{ij}\}$ is a $k \times k$ symmetric zero-diagonal matrix. The joint distribution of $\boldsymbol{\alpha}$ can then be expressed as

$$\boldsymbol{\alpha} \sim N(0, (\mathbf{I} - \theta\mathbf{W})^{-1}\sigma_\epsilon^2\mathbf{I}) \tag{8}$$

Let $\mathbf{D}$ be a diagonal matrix, with each diagonal element being the corresponding row sum of $\mathbf{W}$. We divide each w_{ij} by D_{ii} so that the sum of each row of $\mathbf{W}$ is unity. To ensure $\mathrm{var}(\boldsymbol{\alpha})$ to be symmetric, we set $\sigma_\epsilon^2 \mathrm{I} = \lambda^2\mathbf{D}^{-1}$. The joint distribution of $\boldsymbol{\alpha}$ can then be written as

$$\boldsymbol{\alpha} \sim N(\mathbf{0}, \lambda^2(\mathbf{D} - \theta\mathbf{W})^{-1}) \tag{9}$$

It is shown in the main text that the distribution of $\boldsymbol{y}$ is

$$\mathbf{y} \sim N(\mathbf{x}_i\beta_i + \mathbf{C}\boldsymbol{\gamma}, \mathbf{V}) \tag{10}$$

where $\mathbf{V} = \mathbf{Z}(\mathbf{D} - \theta\mathbf{W})^{-1}\mathbf{Z}'\lambda^2 + \mathbf{I}\sigma_e^2$. For ease of description, let $\mathbf{M}$ denote the matrix $[\mathbf{x}_i \vdots \mathbf{C}]$ and $\boldsymbol{\sigma}$ denote the vector (λ^2, σ_e^2). We can estimate $\{\theta, \boldsymbol{\sigma}\}$ by maximizing the following likelihood function of supplementary Eq. (10)

$$L(\theta, \boldsymbol{\sigma}) = -\frac{1}{2}(\log|\mathbf{V}| + \log|\mathbf{M}'\mathbf{V}^{-1}\mathbf{M}| + \mathbf{Y}'\mathbf{P}\mathbf{Y}) \tag{11}$$

where $\mathbf{V} = \mathbf{Z}(\mathbf{D} - \theta\mathbf{W})^{-1}\mathbf{Z}'\lambda^2 + \mathbf{I}\sigma_e^2$ and $\mathbf{P} = \mathbf{V}^{-1} - \mathbf{V}^{-1}\mathbf{M}(\mathbf{M}'\mathbf{V}^{-1}\mathbf{M})^{-1}\mathbf{M}'\mathbf{V}^{-1}$. In practice, however, estimating $\{\theta, \boldsymbol{\sigma}\}$ requires computing $(\mathbf{D} - \theta\mathbf{W})^{-1}$ iteratively, which is extremely

time-consuming. We note that the precision of $\hat{\theta}$ has little effect on the accuracy of estimating β_i and thus propose to estimate θ by a grid search. We search grid values from 0 to 1 with a step size of 0.1 (by default). For each grid value of $\hat{\theta}$, we use the average-information restricted maximum likelihood (AI-REML) algorithm[6] to estimate $\boldsymbol{\sigma}$. In each AI-REML iteration, $\hat{\boldsymbol{\sigma}}$ is updated as $\hat{\boldsymbol{\sigma}}^{(t+1)} = \hat{\boldsymbol{\sigma}}^{(t)} + \mathbf{J}^{(t)}(\mathbf{A}^{(t)})^{-1}$, where $\mathbf{J}$ is the first derivative of $L(\hat{\theta}, \hat{\boldsymbol{\sigma}})$ with respect to $\hat{\boldsymbol{\sigma}}$

$$\mathbf{J} = \frac{1}{2}\begin{bmatrix} tr\left(\mathbf{PZ}(\mathbf{D} - \hat{\theta}\mathbf{W})^{-1}\mathbf{Z}'\right) - \mathbf{Y}'\mathbf{PZ}(\mathbf{D} - \hat{\theta}\mathbf{W})^{-1}\mathbf{Z}'\mathbf{PY} \\ tr(\mathbf{P}) - \mathbf{Y}'\mathbf{PPY} \end{bmatrix}$$

$\mathbf{A}$ is the average of the observed and expected information matrices

$$\mathbf{A} = \frac{1}{2}\begin{bmatrix} \mathbf{Y}'\mathbf{PZ}(\mathbf{D} - \hat{\theta}\mathbf{W})^{-1}\mathbf{Z}'\mathbf{PZ}(\mathbf{D} - \hat{\theta}\mathbf{W})^{-1}\mathbf{Z}'\mathbf{PY} & \mathbf{Y}'\mathbf{PZ}(\mathbf{D} - \hat{\theta}\mathbf{W})^{-1}\mathbf{Z}'\mathbf{PPY} \\ \mathbf{Y}'\mathbf{PPZ}(\mathbf{D} - \hat{\theta}\mathbf{W})^{-1}\mathbf{Z}'\mathbf{PY} & \mathbf{Y}'\mathbf{PPPY} \end{bmatrix}$$

The $\hat{\boldsymbol{\sigma}}$ that maximizes the likelihood over all the previous grid value of $\hat{\theta}$ is used as the starting value of the AI-REML iterations for the current grid value. For each grid value, the AI-REML iterations are considered as converged when $L(\hat{\theta}, \hat{\boldsymbol{\sigma}})^{t+1} - L(\hat{\theta}, \hat{\boldsymbol{\sigma}})^{t} < 10^{-4}$. The combination of $\hat{\theta}$ and $\hat{\boldsymbol{\sigma}}$ that maximize the likelihood over all the grid values are chosen as the maximum likelihood estimates for θ and $\boldsymbol{\sigma}$.

Given the estimates of $\hat{\theta}$, $\hat{\lambda}^2$, and $\hat{\sigma}_e^2$ from the process above. we estimate β_i and $\boldsymbol{\gamma}$ using the generalized least squares approach:

$$[\beta_i, \boldsymbol{\gamma}] = \left(\mathbf{M}'\hat{\mathbf{V}}^{-1}\mathbf{M}\right)^{-1}\left(\mathbf{M}'\hat{\mathbf{V}}^{-1}\mathbf{Y}\right) \tag{12}$$

where $\hat{\mathbf{V}}^{-1} = \mathbf{Z}\left(\mathbf{D} - \hat{\theta}\mathbf{W}\right)^{-1}\mathbf{Z}'\hat{\lambda}^2 + \mathbf{I}\hat{\sigma}_e^2$.

Section 3. Parameter settings of the cellular deconvolution methods benchmarked in this study

We clustered cells into uniform cell-state bins along the predefined trajectory. To ensure a fair comparison between methods, we used the same cell-state bins in all the cellular deconvolution methods including MeDuSA. We provided the bin labels, reference data, and bulk RNA-seq data to the methods to estimate cell-state abundances. The parameter settings of the methods are summarized as follows:

1) For CPM, we downloaded the R package of scBio (v0.1.5) and used the default parameters of the CPM function, including "neighborhoodSize = 10, modelSize = 50, minSelection = 5". The resulting output of CPM provides abundance estimations for individual cells. To obtain estimates of cell-state abundance, we calculated the average abundance of cells within each

cell-state bin.

2) For CIBERSORT, we downloaded the released R source code (v1.04) and built gene expression profiles (GEPs) by averaging gene expression profiles of cells in each cell-state bin. We used the signature genes selected by MeDuSA.

3) For BayesPrism, we downloaded the R package of BayesPrism (v2.0) and filtered out genes of chrX, chrY, mitochondria, and ribosome. We constructed the BayesPrism object using the function of "new.prism" with parameters of "outlier.cut=0.01, outlier.fraction=0.1" and used the functions of "run.prism" and "get.fraction" to obtain abundance estimations.

4) For Scaden, we downloaded the python package of scaden (v1.1.2) and generated pseudo-bulk data with settings of 500 cells per pseudo-bulk sample and 5,000 samples in total. Based on the pseudo-bulk data, we trained the model using a batch size of 128, a learning rate of 1.00E-4, and 5,000 steps. We used the command of "predict" to obtain abundance estimations.

5) For TAPE, we downloaded the python package of TAPE (v1.1.0) and used the function of "Deconvolution" with parameters of "variance threshold=0.98, batch size=128, epochs=500".

6) For MuSiC, we downloaded the R package of MuSiC (v1.0.0) and ran it using the default settings. MuSiC states that it takes advantage of multiple subjects in the reference data to improve accuracy. In simulations, we randomly assigned datasets not generated from multiple subjects to two different subjects, as done in a previous study[20]. In the real-data benchmark analysis, we used reference scRNA-seq data generated from multiple donors and provided the donor labels to MuSiC.

7) For ssGSEA, we downloaded the R package of GSVA (v1.46) and used the function of "FindMarkers" of the Seurat package to identify signature genes (the top 5 genes with the lowest p-values) of each cell state. To ensure a fair comparison in the benchmark analysis, the ssGSEA output scores were scaled to fractional abundances between 0 and 1.

Section 4. Processing the scRNA-seq data

Raw scRNA-seq reads were mapped to the reference genome using Cell Ranger. Quality control was conducted by visual inspection of the quality control plots using Seurat V3[21]. Low-quality cells were removed according to the following criteria: 1) cells with >10% mitochondrial counts, and 2) cells with >4500 and <200 expressed genes (potential duplets or empty droplets). The cells retained after filtering were normalized and corrected for confounding factors such as the number of UMIs and percentage of mitochondrial content. To cluster cells, principal component analysis (PCA) was applied to the top 2,000 high cell-to-

cell variation genes identified by the "FindVariableFeatures" function in Seurat. The number of PCs used for cell clustering was determined by visual inspection of the elbow and jackstraw plots (using 15-20 PCs). The functions "FindNeighbors & FindClusters" in Seurat were employed to cluster cells based on the selected PCs with the resolution parameter set to [0.5-2]. Cell type identifications were obtained from the previous studies (when available) and validated in this study as follows: 1) assigning each cell to a specific cell type according to its gene expression similarity to the Human Primary Cell Atlas[22] using SingleR[23]; 2) selecting cells with discordant cell type labels; 3) finalizing cell type annotations based on the expression levels of known marker genes. Cell trajectory analysis were performed using Slingshot[24], CytoTRACE[25], or scVelo[26]. Additional processing steps for specific datasets were summarized as follows.

Esophagus scRNA-seq data (PRJEB31843): The esophagus scRNA-seq data from Madissoon et al. profiled cell-transcriptomics of 24 esophagi using the 10X Genomics 3' v2 protocol[27]. We included data from healthy esophagi at time points 0 h, 12 h and 24 h ($n = 15$), as the quality of the scRNA-seq data remained stable during 24 hours of cold storage.

Bone marrows scRNA-seq data (GSE120221): We downloaded the count data[28] and filtered out doublets using DoubletFinder[29]. Based on the expression levels of marker genes, we classified cells into six major cell types, including HSPCs (*AVP*), B cells (*CD79A* and *CD79B*), T cells (*CD3G* and *CD3E*), NK cells (*NKG7*), monocytes (*SPI1*), and erythrocytes (*HBD*). We extracted data for monocytes and annotated the development trajectory as described above.

iPSC scRNA-seq data (ERP01600015): Lappalainen et al.[30] conducted bulk RNA-seq analysis on iPSCs, and Cuomo et al.[31] generated scRNA-seq data using the same cell lines. Cuomo et al.[32] showed a high level of consistency between the two datasets on day 0 of cultivation. Therefore, for our benchmark analysis, we utilized the scRNA-seq and bulk RNA-seq data from day 0 of cultivation. To ensure accurate measurements of cell-state abundance in the scRNA-seq data, we included samples with a minimum of 200 cells, resulting in the inclusion of six samples. We used CytoTRACE to annotate the differentiation trajectory of iPSCs.

hPSC scRNA-seq data (GSE75748): Chu et al.[33] performed scRNA-seq and bulk RNA-seq of hPSCs, with the bulk RNA-seq data collected from multiple technical replicates. We used the mean of the technical replicates to reduce noise in the data. The sample with only 69 cells

("TB") was removed. We used CytoTRACE to annotate the hPSC differentiation trajectory.

COVID-19 scRNA-seq data (GSE152418): We processed a set of scRNA-seq data from COVID-19 PBMC[7]. The cell type labels of this data were obtained from the prior work and validated in this study using the pipeline described above. Following the previous studies[34,35], we adjusted for the potential confounding factors in this data including the "ribosomal protein genes (gene symbol pattern: ^RP([0-9]+-|[LS]))" and "cell disassociation induced genes[35]" using the Seurat function "AddModuleScore".

Melanoma scRNA-seq data (GSE77940): The scRNA-seq data of melanoma patients generated using Smart-Seq2 was obtained from Tirosh et al.[36]. Cell type identifications were provided by Tirosh et al. and confirmed by us as described above. We further annotated CD4+ and CD8+ T cells according to the following criteria[37]: 1) cells expressing *CD4* but not *CD8A* nor *CD8B* were assigned to CD4+ T cells; 2) cells expressing *CD8A* or *CD8B* but not *CD4* were assigned to CD8+ T cells. T cells that did not belong to these cell types were removed. The exhaustion score was obtained based on the gene set provided by Tirosh et al.[36] (computed as the mean expression level of exhaustion gene set minus the mean expression level of a naïve gene set).

Section 5. Processing the bulk RNA-seq data

Counts/FPKM/TPM matrices of these data were obtained from the prior studies or public databases. When the processed expression matrices were unavailable, we collected the FASTQ reads and obtained the gene expression matrix according to the following procedure: 1) raw RNA-seq FASTQ reads were cleaned using fastp[38] to remove adapters or low-quality reads; 2) the remaining reads were aligned to the human reference genome (GRCh38) and summarized to counts data using STAR[39]; 3) summary counts were transformed to TPM using RSEM[40] where necessary. The expansion level of the T cell receptor (TCR) of TCGA melanoma bulk RNA-seq data were estimated using MiXCR[41].

Section 6. Processing the scATAC-seq data

We collected the esophagus scATAC-seq data ($n = 3$) from a previous study[42] and followed the standard pipelines to process the scATAC-seq data. We started with generating the snap file from FASTQ reads using snaptools[43]. Based on the snap file, we created the cell-by-bin matrix by dividing the genome into 5-kb consecutive windows. We filtered out the bins overlapping with the mitochondria, ENCODE blacklist[44], chromosome X, chromosome Y, and

ambiguous chromosomes, as well as the top 5% bins that overlap with the invariant features (e.g., promoters of housekeeping gene). The subsequent dimensionality reduction analysis was performed using the nonlinear diffusion map algorithm[45] embedded in the function "runDiffusionMaps" of the SnapATAC package[43]. We inspected the first 50 eigenvectors and selected 20 eigenvectors to construct the k-nearest neighbor graph ($k = 15$) using the function "runKNN" and batch correction among samples using the Harmony method[46]. We then performed cell clustering using the Leiden algorithm[47] with a resolution of 0.5 and visualized the cell clusters using UMAP. We removed clusters with fewer than 100 cells. Cell types of the remaining clusters were then annotated based on the enrichment of promoter accessibilities ($\pm$1Kb around TSS) of the marker genes[42]. For each cluster, peak calling was performed on Tn5-corrected insertions using MACS2[48] with parameters "–nomodel –shift 100 –ext 200 –qval 5e-2 –B –SPMR –keep-dup all". We then aggregated peaks from all clusters to generate a union peak list, based on which we generated the cell-by-peak matrix.

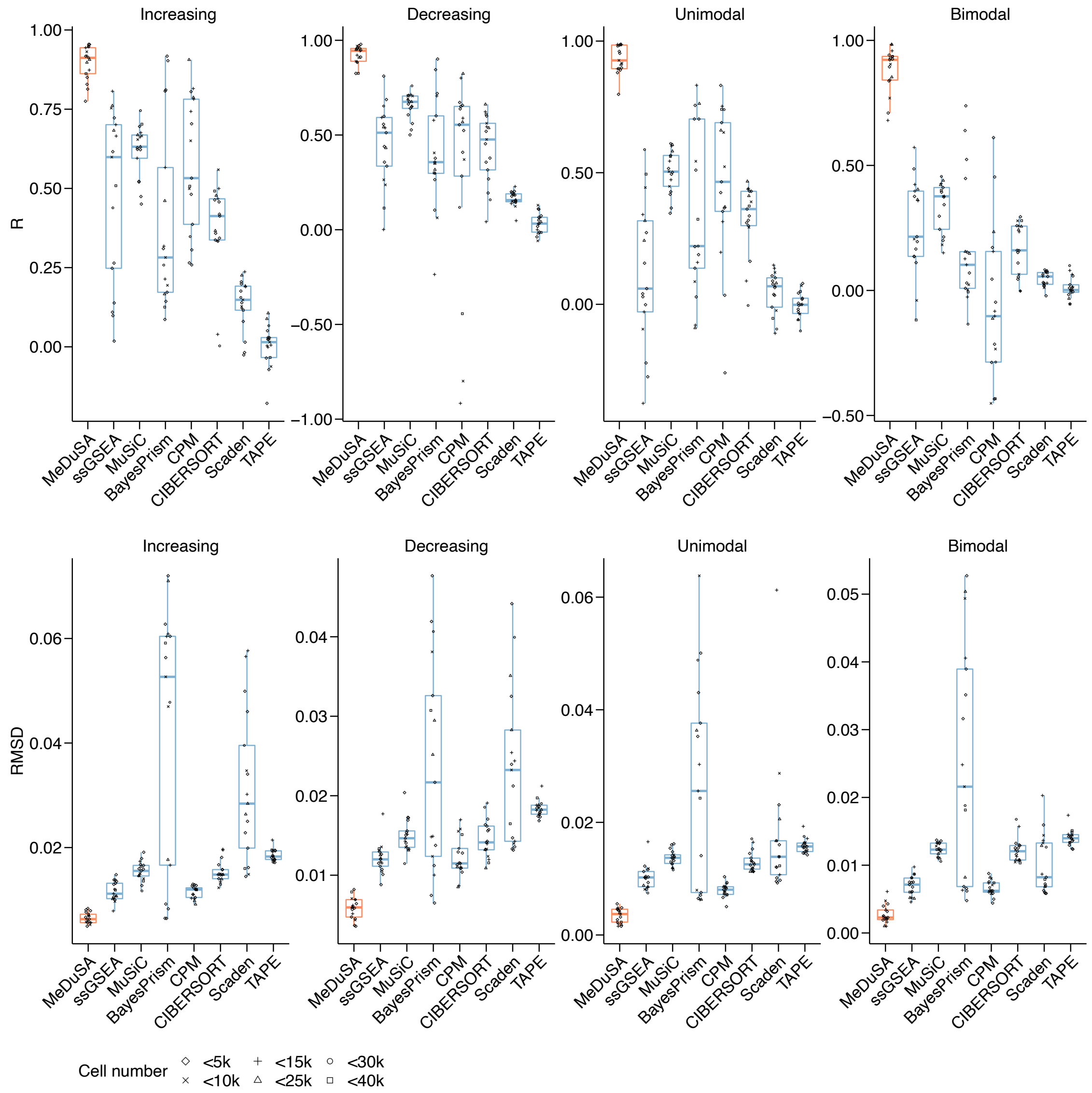

Supplementary Figure 1. Benchmarking the cellular deconvolution methods by simulations. Boxplot of R (the higher the better) and RMSD (the lower the better) for each deconvolution method. Each dot represents the mean deconvolution accuracy over five replicates for a simulation source dataset. The box indicates the interquartile range (IQR), the line within the box represents the median value, and the whiskers extend to data points within 1.5 times the IQR.

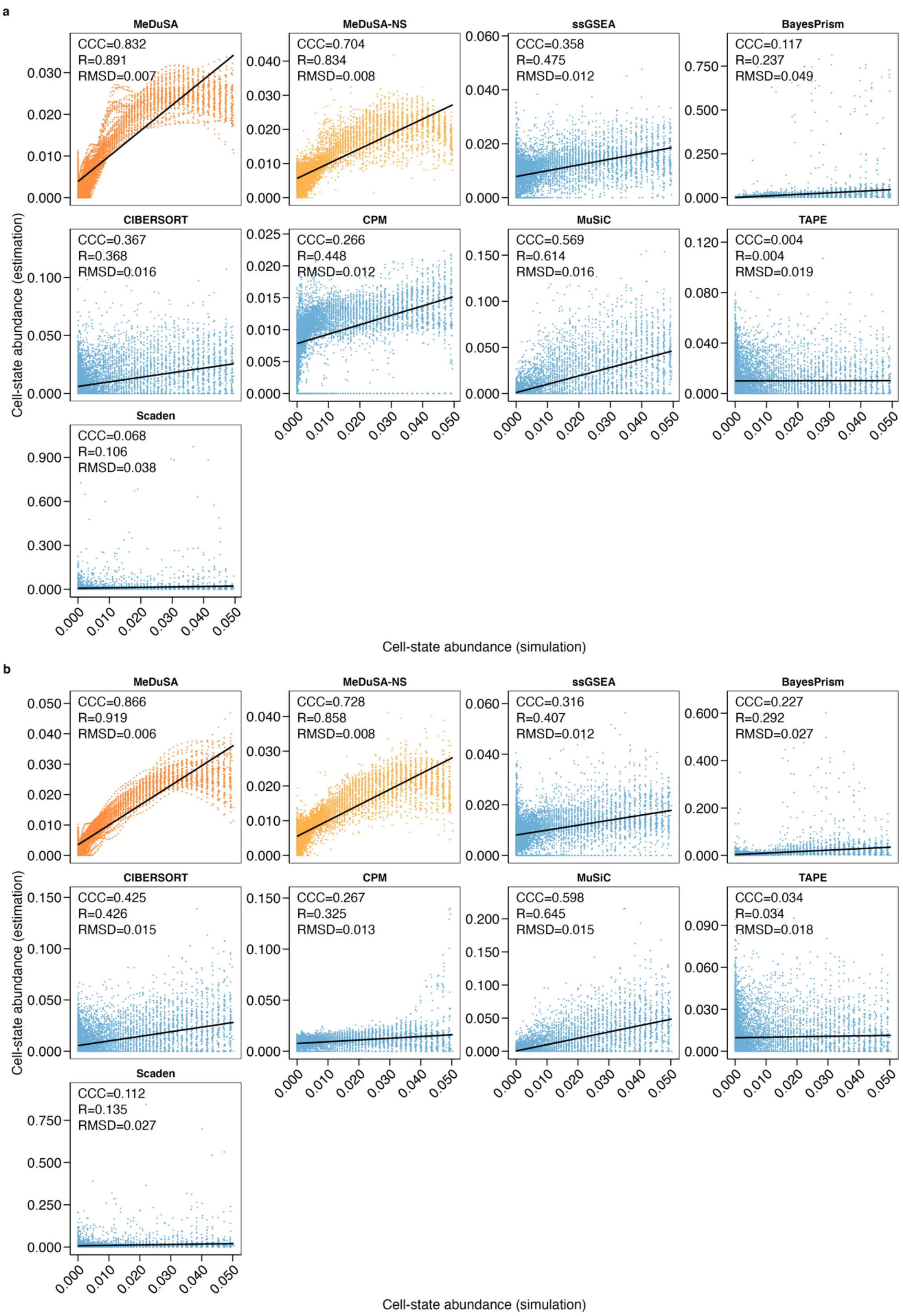

a
MeDuSA
CCC=0.832
R=0.891
RMSD=0.007
MeDuSA-NS
CCC=0.704
R=0.834
RMSD=0.008
ssGSEA
CCC=0.358
R=0.475
RMSD=0.012
BayesPrism
CCC=0.117
R=0.237
RMSD=0.049
CIBERSORT
CCC=0.367
R=0.368
RMSD=0.016
CPM
CCC=0.266
R=0.448
RMSD=0.012
MuSiC
CCC=0.569
R=0.614
RMSD=0.016
TAPE
CCC=0.004
R=0.004
RMSD=0.019
Scaden
CCC=0.068
R=0.106
RMSD=0.038
Cell-state abundance (estimation)
Cell-state abundance (simulation)
b
MeDuSA
CCC=0.866
R=0.919
RMSD=0.006
MeDuSA-NS
CCC=0.728
R=0.858
RMSD=0.008
ssGSEA
CCC=0.316
R=0.407
RMSD=0.012
BayesPrism
CCC=0.227
R=0.292
RMSD=0.027
CIBERSORT
CCC=0.425
R=0.426
RMSD=0.015
CPM
CCC=0.267
R=0.325
RMSD=0.013
MuSiC
CCC=0.598
R=0.645
RMSD=0.015
TAPE
CCC=0.034
R=0.034
RMSD=0.018
Scaden
CCC=0.112
R=0.135
RMSD=0.027
Cell-state abundance (estimation)
Cell-state abundance (simulation)

c

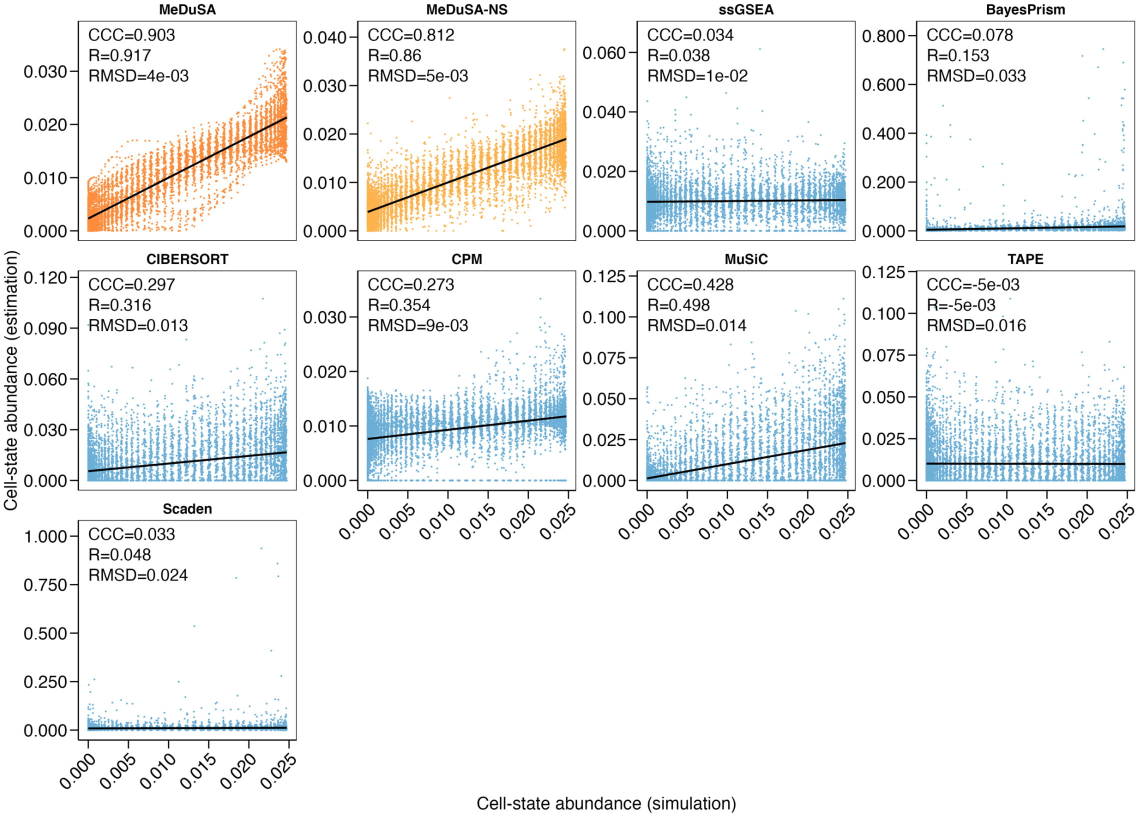
MeDuSA
CCC=0.903
R=0.917
RMSD=4e-03
MeDuSA-NS
CCC=0.812
R=0.86
RMSD=5e-03
ssGSEA
CCC=0.034
R=0.038
RMSD=1e-02
BayesPrism
CCC=0.078
R=0.153
RMSD=0.033
CIBERSORT
CCC=0.297
R=0.316
RMSD=0.013
CPM
CCC=0.273
R=0.354
RMSD=9e-03
MuSiC
CCC=0.428
R=0.498
RMSD=0.014
TAPE
CCC=-5e-03
R=-5e-03
RMSD=0.016
Scaden
CCC=0.033
R=0.048
RMSD=0.024
Cell-state abundance (estimation)
Cell-state abundance (simulation)

d

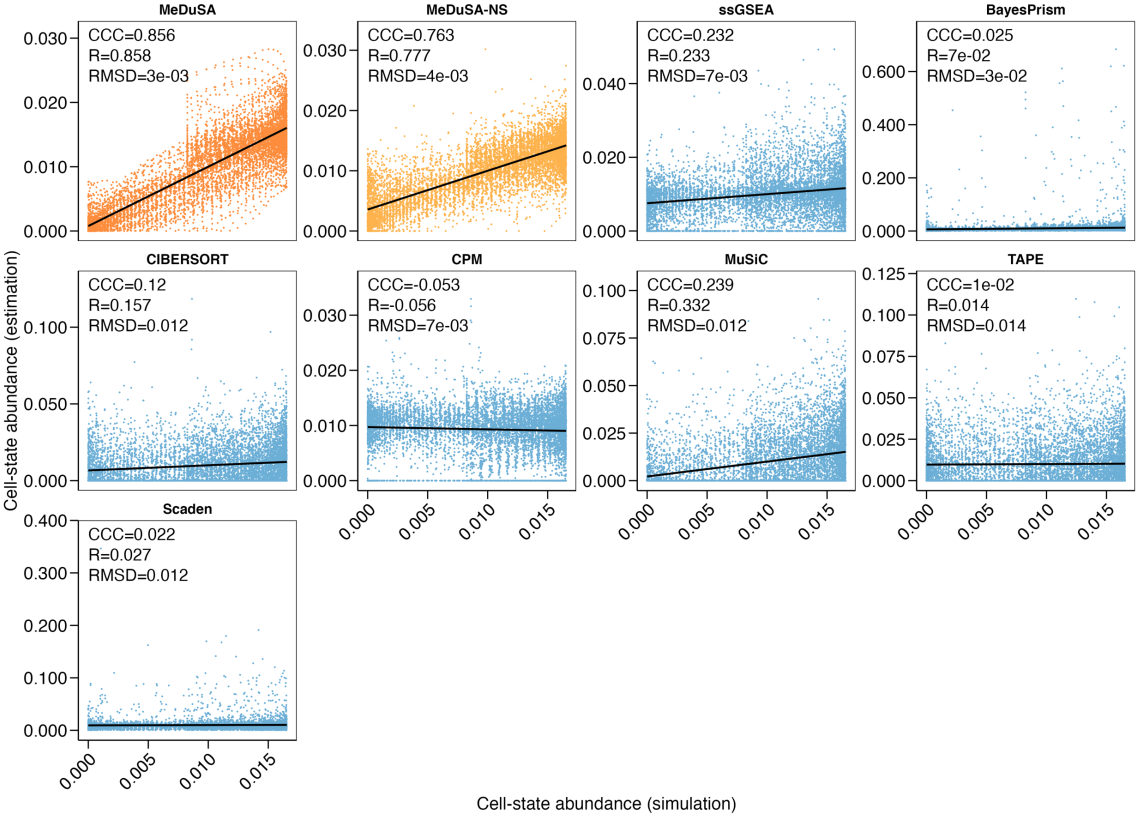
MeDuSA
CCC=0.856
R=0.858
RMSD=3e-03
MeDuSA-NS
CCC=0.763
R=0.777
RMSD=4e-03
ssGSEA
CCC=0.232
R=0.233
RMSD=7e-03
BayesPrism
CCC=0.025
R=7e-02
RMSD=3e-02
CIBERSORT
CCC=0.12
R=0.157
RMSD=0.012
CPM
CCC=-0.053
R=-0.056
RMSD=7e-03
MuSiC
CCC=0.239
R=0.332
RMSD=0.012
TAPE
CCC=1e-02
R=0.014
RMSD=0.014
Scaden
CCC=0.022
R=0.027
RMSD=0.012
Cell-state abundance (estimation)
Cell-state abundance (simulation)

Supplementary Figure 2. Scatter plot comparing the estimated cell-state abundance to the simulated ground truth. The scatter plot combines the results from multiple datasets and simulation replicates. The evaluation metrics (CCC, R, and RMSD) were computed based on the pooled results. The x-axis represents the simulated cell-state abundance, and the y-axis represents the estimated cell-state abundance. Panels (a-d) depict the pre-designed cell-state abundance distributions of (a) increasing, (b) decreasing, (c) unimodal, and (d) bimodal patterns, respectively.

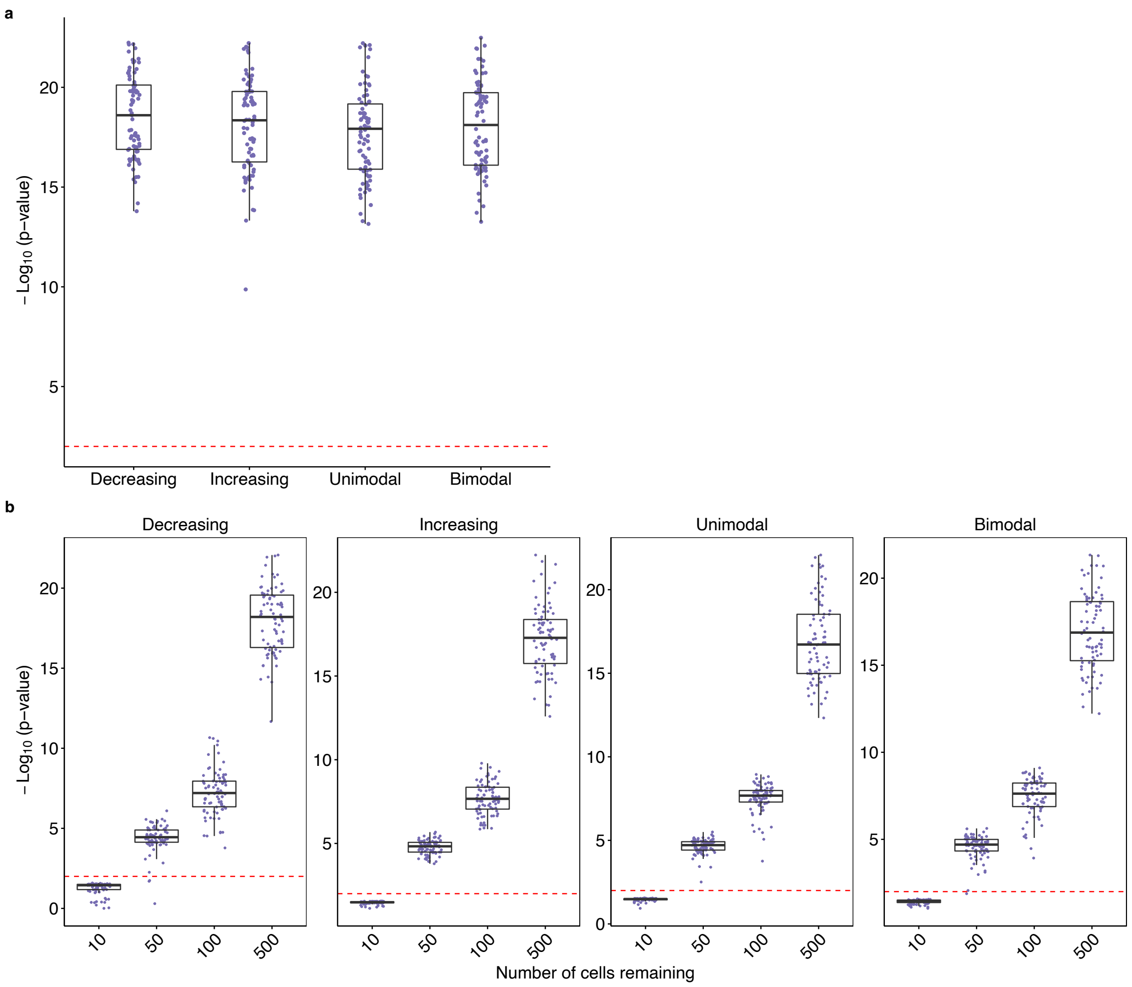

Supplementary Figure 3. Boxplots of p-values for the random-effect component of the MeDuSA from the analyses of the simulation data. (a) P-values from analyses with all the remaining cells fitted in the random-effect model. The x-axis represents the pre-designed cell-state abundance distributions, and the y-axis is the -log10(p-value). (b) P-values from analyses with different numbers of cells fitted in the random-effect component. We grouped cells of the focal cell type into ten uniformly distributed cell bins over the cell-state trajectory and randomly sampled a subset of cells from each cell bin to be fitted in the random-effect component. The p-values of the random-effect component were computed using a one-sided chi-squared test. Each dot represents -log10(p-value) for one simulation replicate from one source data (total $n = 80$). The box indicates the IQR, the line within the box represents the median value, and the whiskers extend to data points within 1.5 times the IQR.

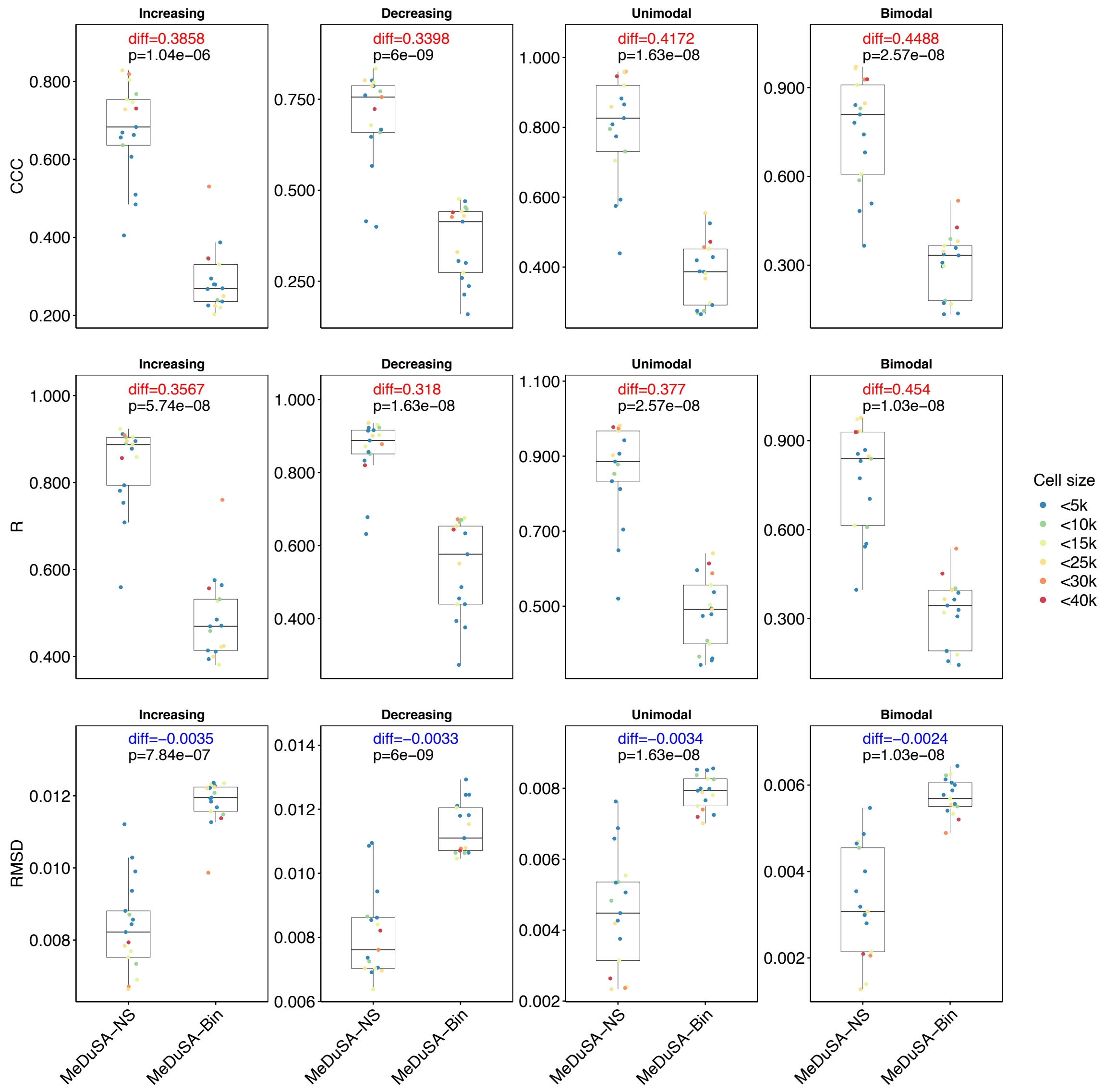

Supplementary Figure 4. Deconvolution accuracy of MeDuSA with the means of cell bins fitted as random effects. We grouped cells of the focal cell type into bins along the cell-state trajectory and fitted the means of each bins in the random-effect components (referred to as MeDuSA-Bin). Each dot represents the mean deconvolution accuracy over five replicates for one simulation source data, colored by the number of cells in the data. The box indicates the interquartile IQR, the line within the box represents the median value, and the whiskers extend to data points within 1.5 times the IQR. The p-values were calculated using the two-sided Wilcoxon test.

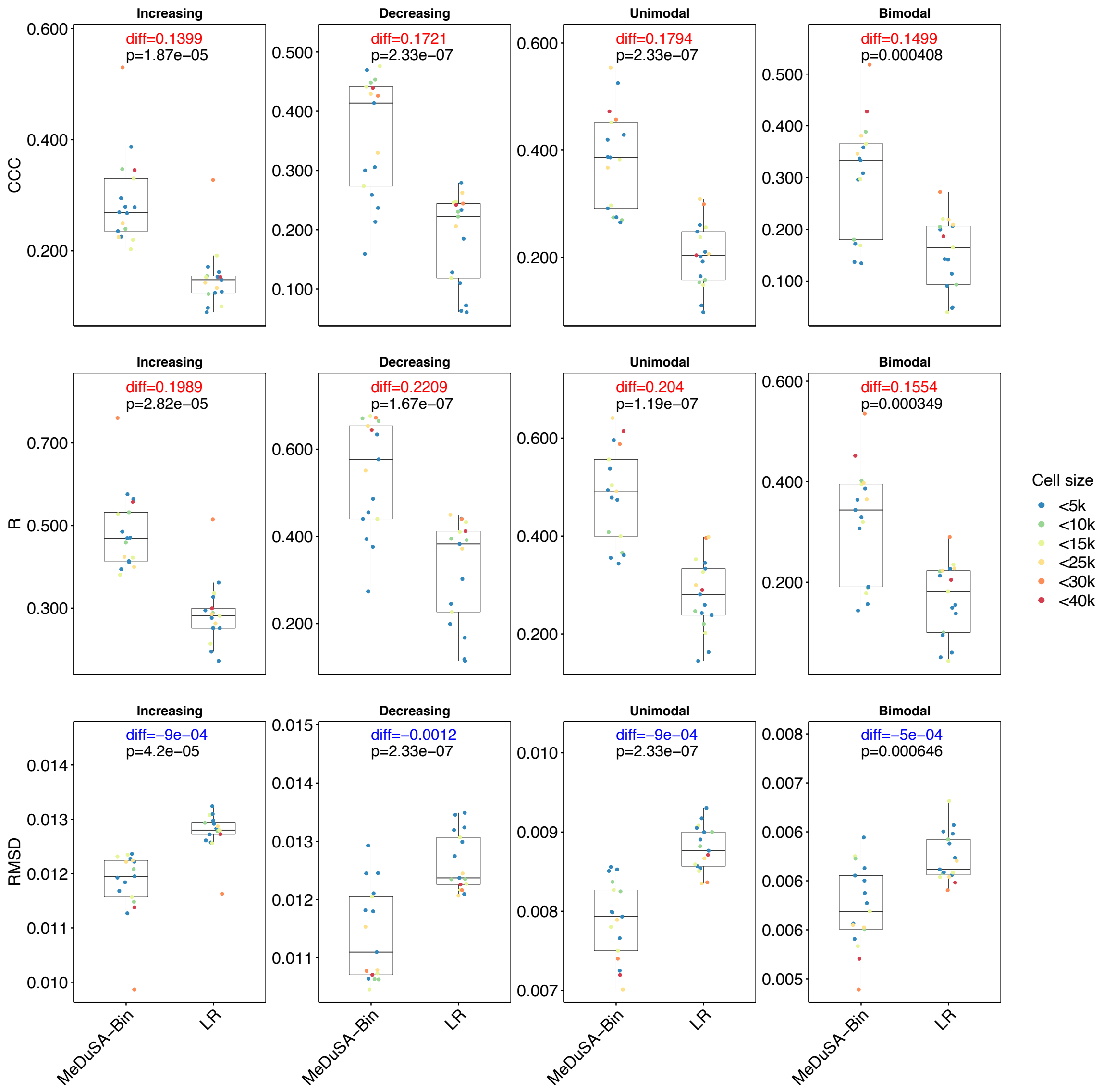

Supplementary Figure 5. Deconvolution accuracy of MeDuSA with the means of cell bins fitted as fixed effects. We grouped cells of focal cell type into bins along the cell-state trajectory and fitted the mean of each cell bin as a fixed effect (referred to as linear regression or LR). Each dot represents the mean deconvolution accuracy over five replicates for one simulation source data, colored by the number of cells in the data. The box indicates the interquartile IQR, the line within the box represents the median value, and the whiskers extend to data points within 1.5 times the IQR. The p-values were calculated using the two-sided Wilcoxon test.

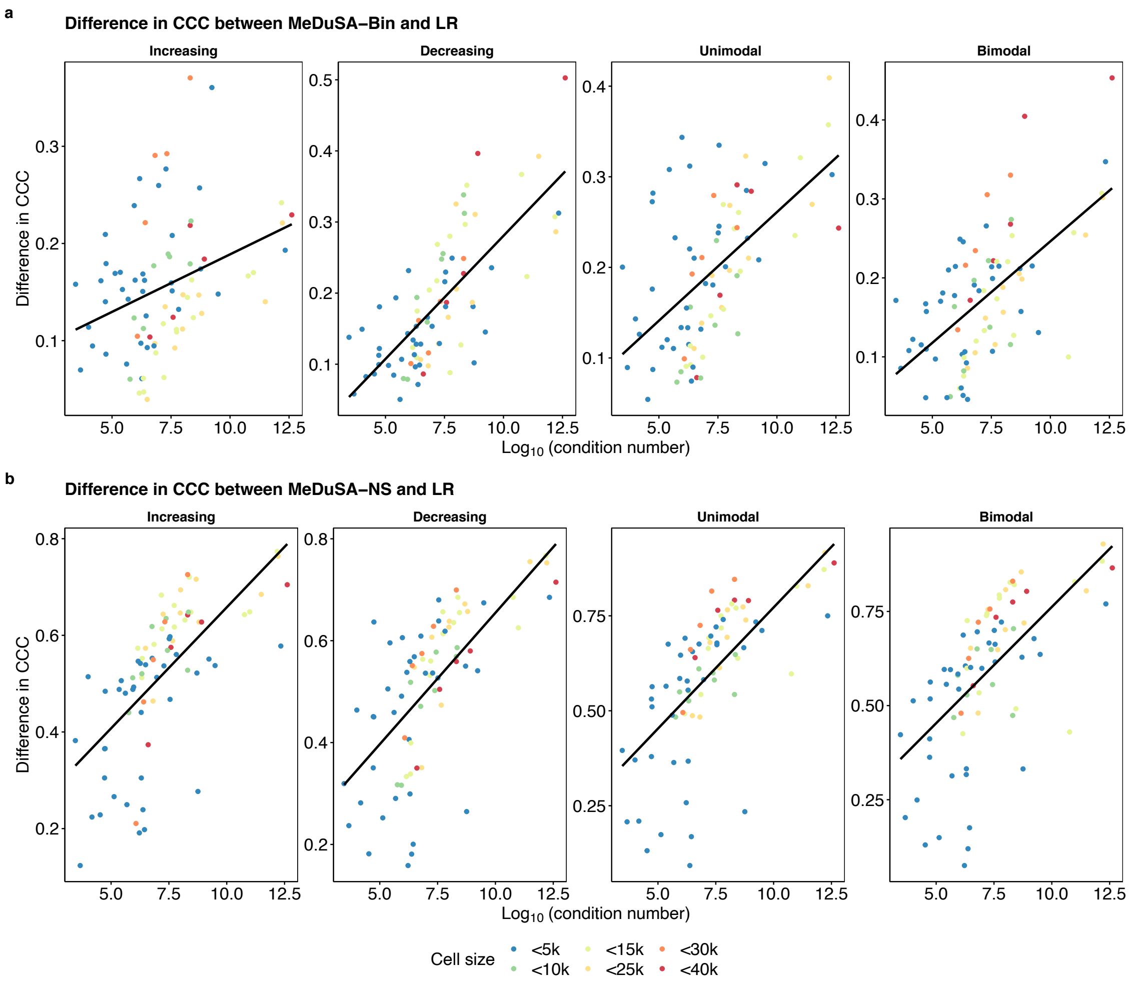

Supplementary Figure 6. Differences in deconvolution accuracy (CCC) between MeDuSA-NS, MeDuSA-Bin, and LR. (a) The change of the difference in CCC between MeDuSA-Bin and LR with the condition number (a metric to measure the level of collinearity between cell states). (b) The change of the difference in CCC between MeDuSA-NS and LR with the condition number. Note that in MeDuSA-NS the mean of each focal cell state is fitted as a fixed effect with the remaining cells fitted individually in the random-effect component. Each point shows the mean deconvolution accuracy over five replicates for one simulation source data, colored by the number of cells in the data.

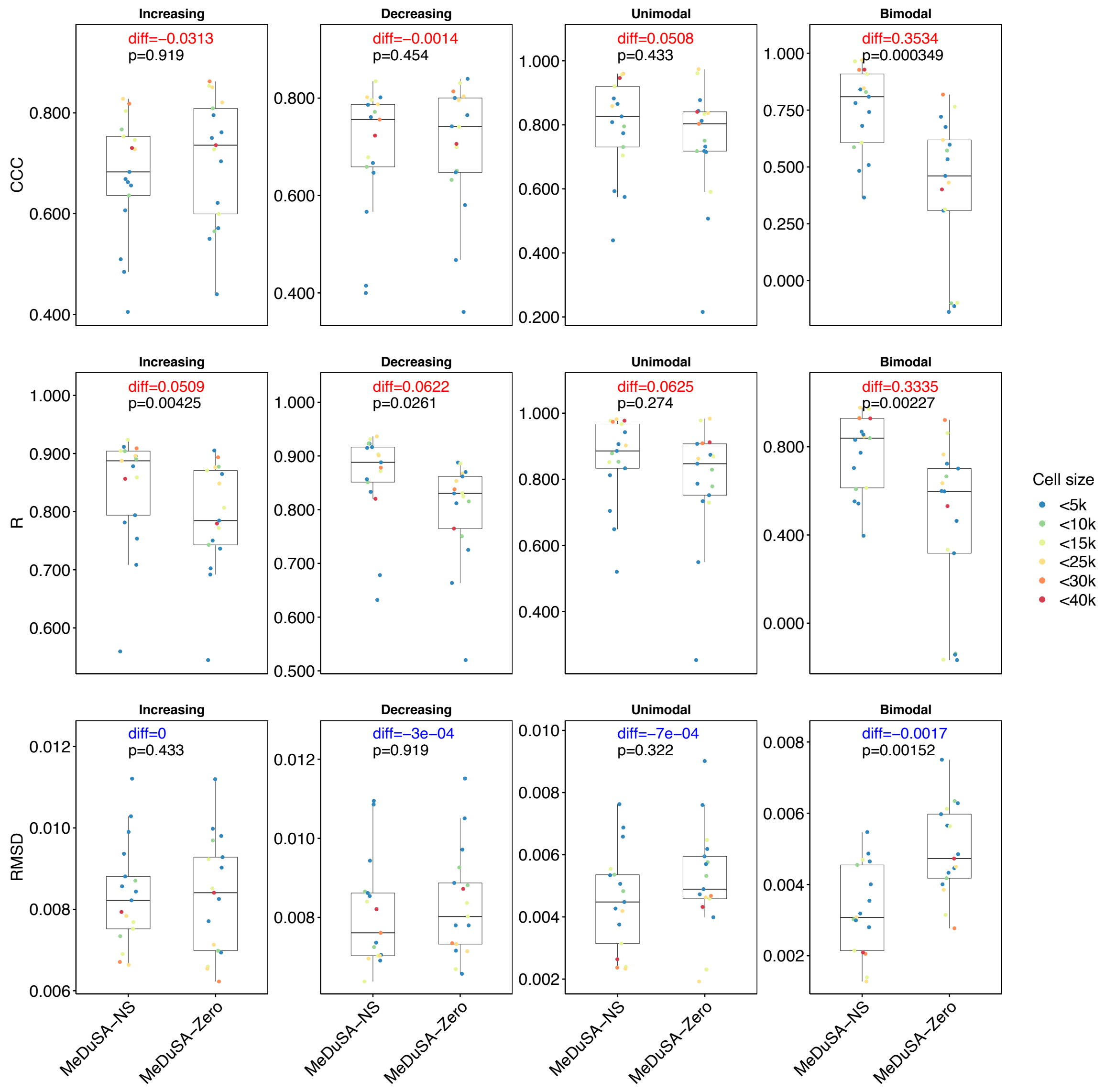

Supplementary Figure 7. Deconvolution accuracy of MeDuSA without modeling cell correlations. We constrained the cell correlations in the MeDuSA model to zero (referred to as MeDuSA-Zero). Each dot represents the mean deconvolution accuracy over five replicates for one simulation source data, colored by the number of cells in the data. The box indicates the interquartile IQR, the line within the box represents the median value, and the whiskers extend to data points within 1.5 times the IQR. The p-values were calculated using the two-sided Wilcoxon test.

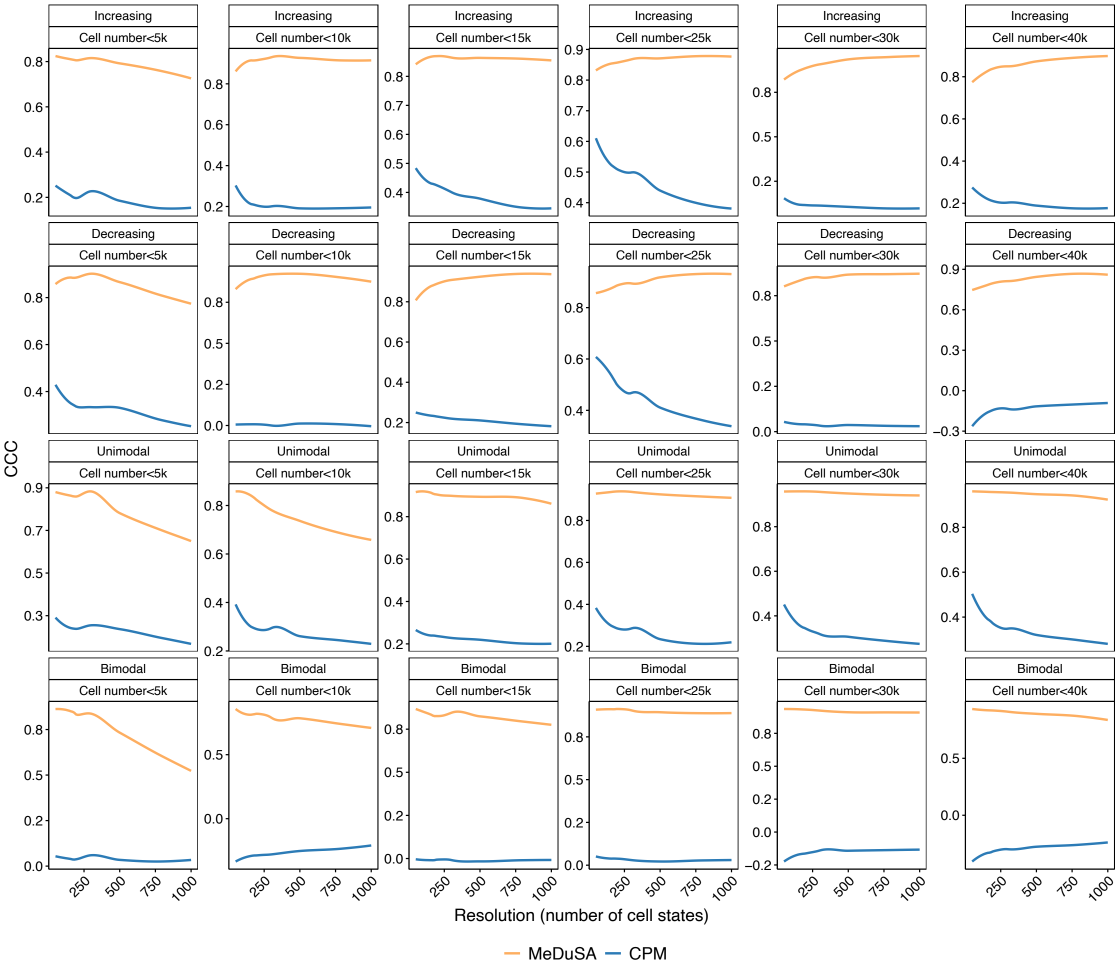

Supplementary Figure 8. Deconvolution accuracy of MeDuSA and CPM with different deconvolution resolutions. The x-axis represents deconvolution resolution (i.e., the number of cell state bins). The y-axis represents the deconvolution accuracy (CCC). Each line shows the mean accuracy over simulation replicates, colored by methods. Subtitles show the number of cells in the simulation source data and the simulated cell-state abundance distribution.

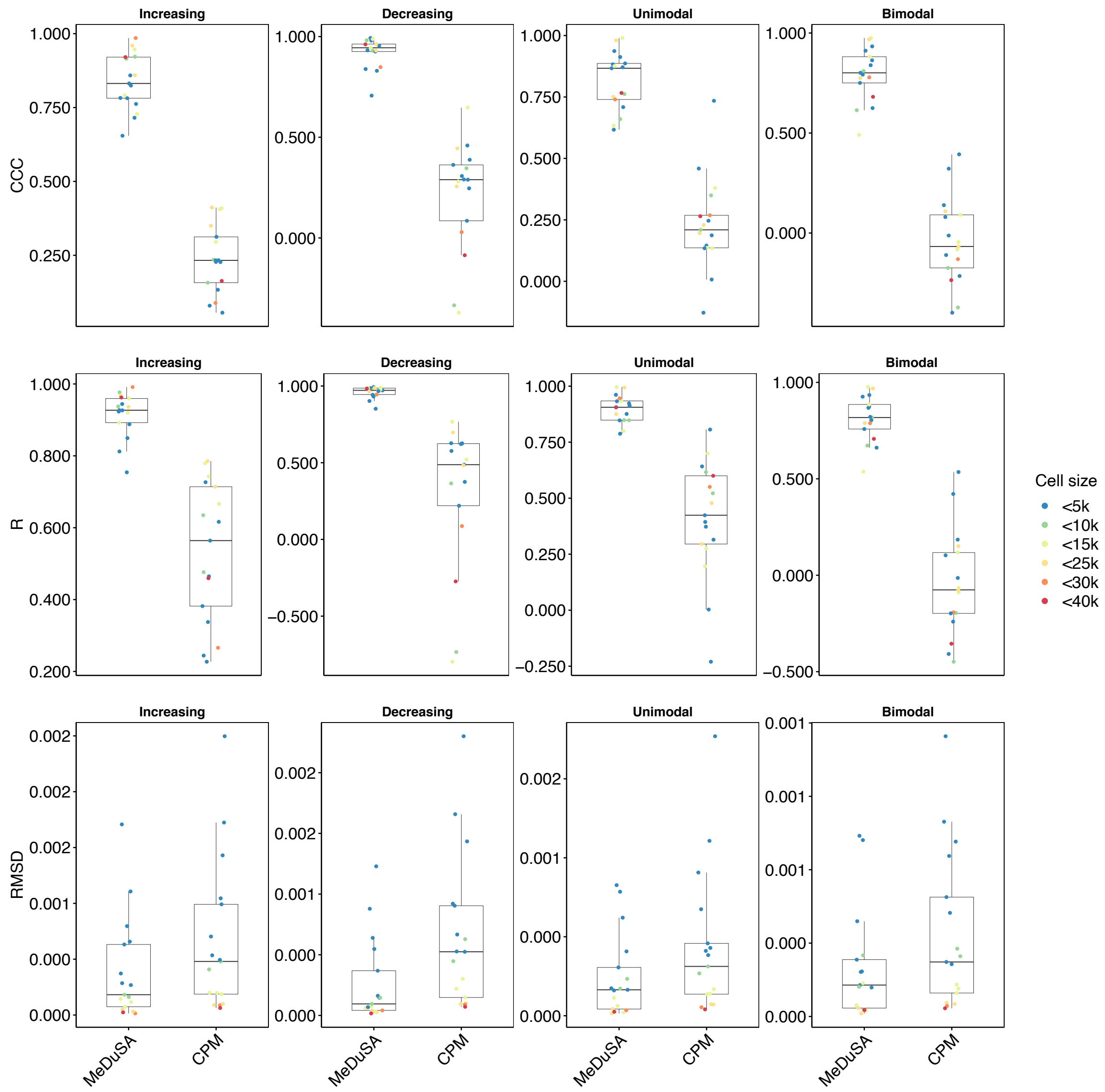

Supplementary Figure 9. Deconvolution accuracy of MeDuSA and CPM at the single cell resolution. Each dot represents the mean deconvolution accuracy over five replicates for one simulation source data, colored by the number of cells in the data. The box indicates the interquartile IQR, the line within the box represents the median value, and the whiskers extend to data points within 1.5 times the IQR.

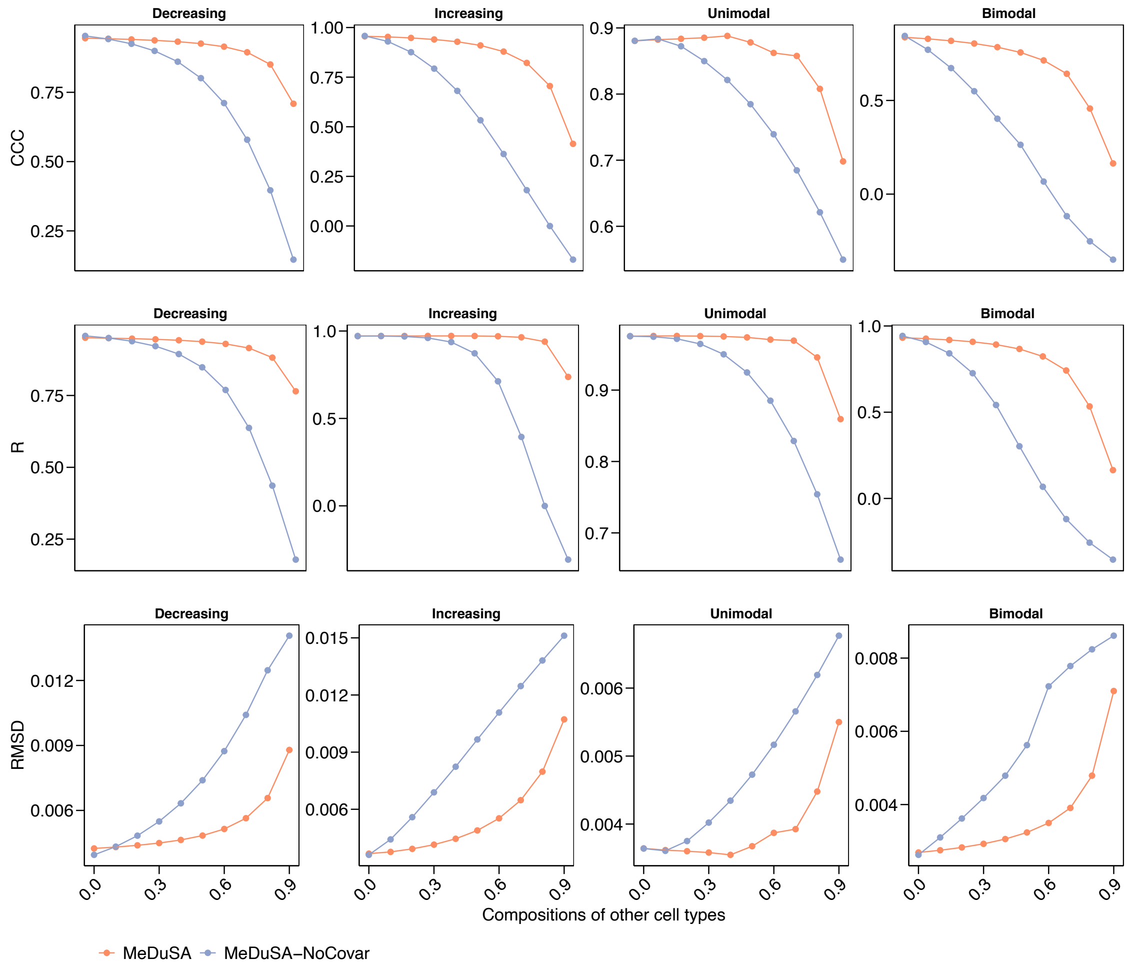

Supplementary Figure 10. Including cell type covariates improves the robustness of MeDuSA. This simulation analysis was performed using the esophagus data. To assess the effects of other cell types on the performance of MeDuSA, we varied the compositions of the other cell types from 0 to 0.9. The compositions of the other cell types was randomly generated from $U(0,1)$ and then normalized to sum to one minus the composition of the focal cell type (i.e., epithelial cells). The x-axis indicates the compositions of other cell types, and the y-axis shows the deconvolution accuracy. The MeDuSA model without cell type covariates is denoted as MeDuSA-NoCovar.

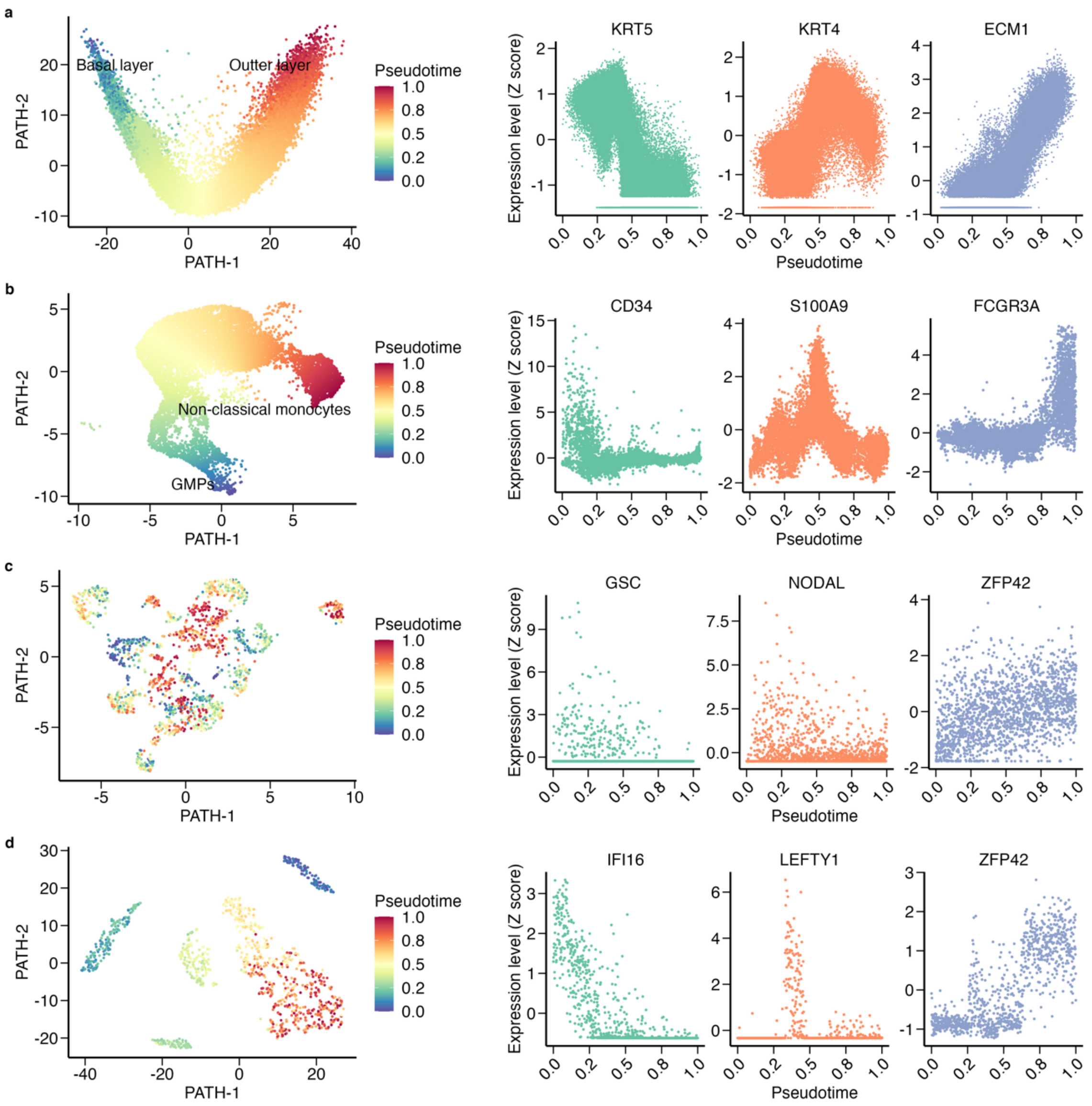

Supplementary Figure 11. The datasets used in the real-data benchmark analysis. (a-d) The sample-matched bulk RNA-seq and scRNA-seq datasets from (a) human esophagus, (b) human bone marrows, (c) iPSCs, and (d) hPSCs. In each panel, the left figures show the pseudotime, and the right figures show the expression pattern of marker genes along the pseudotime. Details of each dataset are presented in the Section 4 of the Supplementary Note.

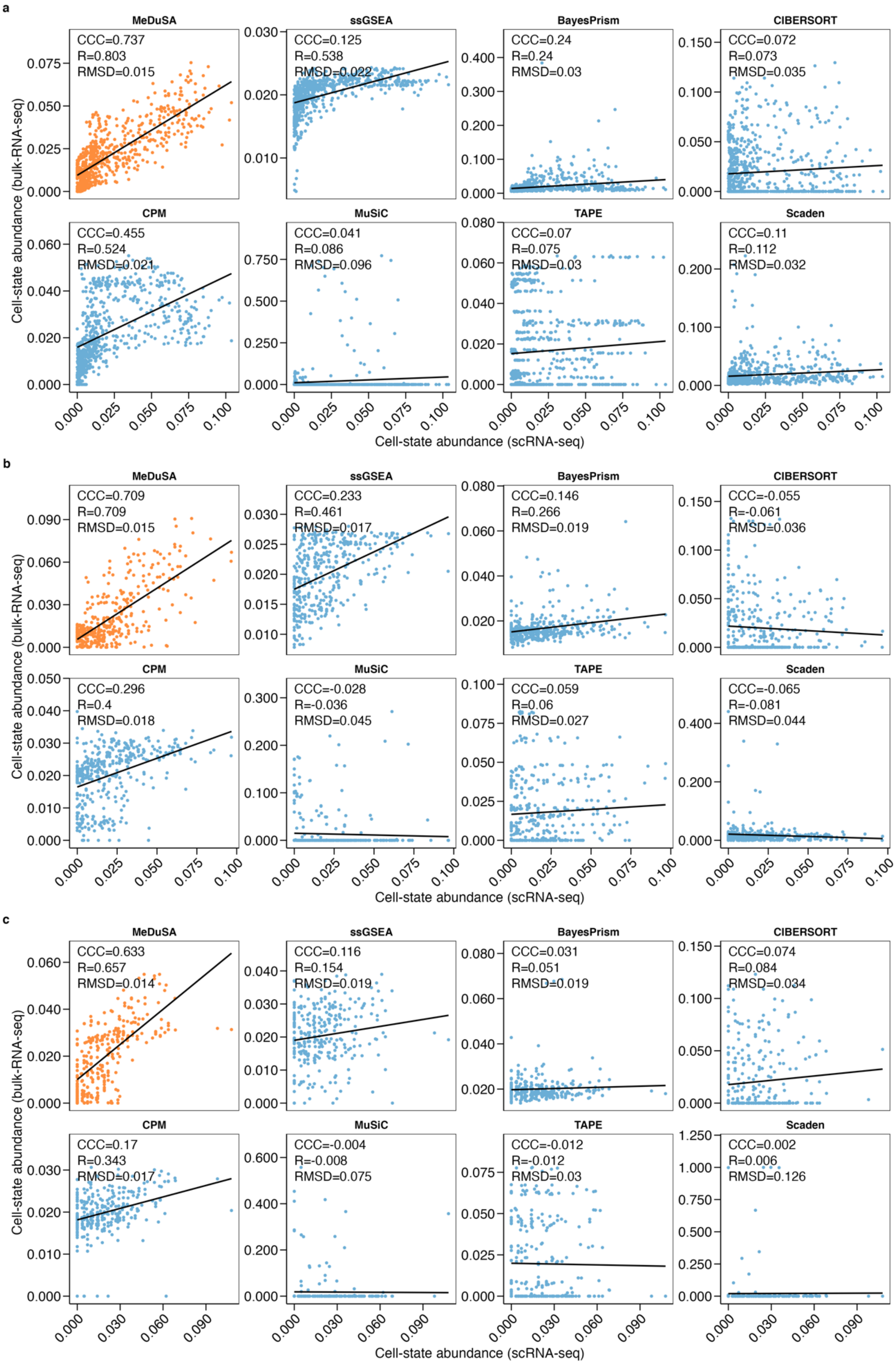
a
MeDuSA
CCC=0.737
R=0.803
RMSD=0.015
ssGSEA
CCC=0.125
R=0.538
RMSD=0.022
BayesPrism
CCC=0.24
R=0.24
RMSD=0.03
CIBERSORT
CCC=0.072
R=0.073
RMSD=0.035
CPM
CCC=0.455
R=0.524
RMSD=0.021
MuSiC
CCC=0.041
R=0.086
RMSD=0.096
TAPE
CCC=0.07
R=0.075
RMSD=0.03
Scaden
CCC=0.11
R=0.112
RMSD=0.032
Cell-state abundance (bulk-RNA-seq)
Cell-state abundance (scRNA-seq)
b
MeDuSA
CCC=0.709
R=0.709
RMSD=0.015
ssGSEA
CCC=0.233
R=0.461
RMSD=0.017
BayesPrism
CCC=0.146
R=0.266
RMSD=0.019
CIBERSORT
CCC=-0.055
R=-0.061
RMSD=0.036
CPM
CCC=0.296
R=0.4
RMSD=0.018
MuSiC
CCC=-0.028
R=-0.036
RMSD=0.045
TAPE
CCC=0.059
R=0.06
RMSD=0.027
Scaden
CCC=-0.065
R=-0.081
RMSD=0.044
Cell-state abundance (bulk-RNA-seq)
Cell-state abundance (scRNA-seq)
c
MeDuSA
CCC=0.633
R=0.657
RMSD=0.014
ssGSEA
CCC=0.116
R=0.154
RMSD=0.019
BayesPrism
CCC=0.031
R=0.051
RMSD=0.019
CIBERSORT
CCC=0.074
R=0.084
RMSD=0.034
CPM
CCC=0.17
R=0.343
RMSD=0.017
MuSiC
CCC=-0.004
R=-0.008
RMSD=0.075
TAPE
CCC=-0.012
R=-0.012
RMSD=0.03
Scaden
CCC=0.002
R=0.006
RMSD=0.126
Cell-state abundance (bulk-RNA-seq)
Cell-state abundance (scRNA-seq)

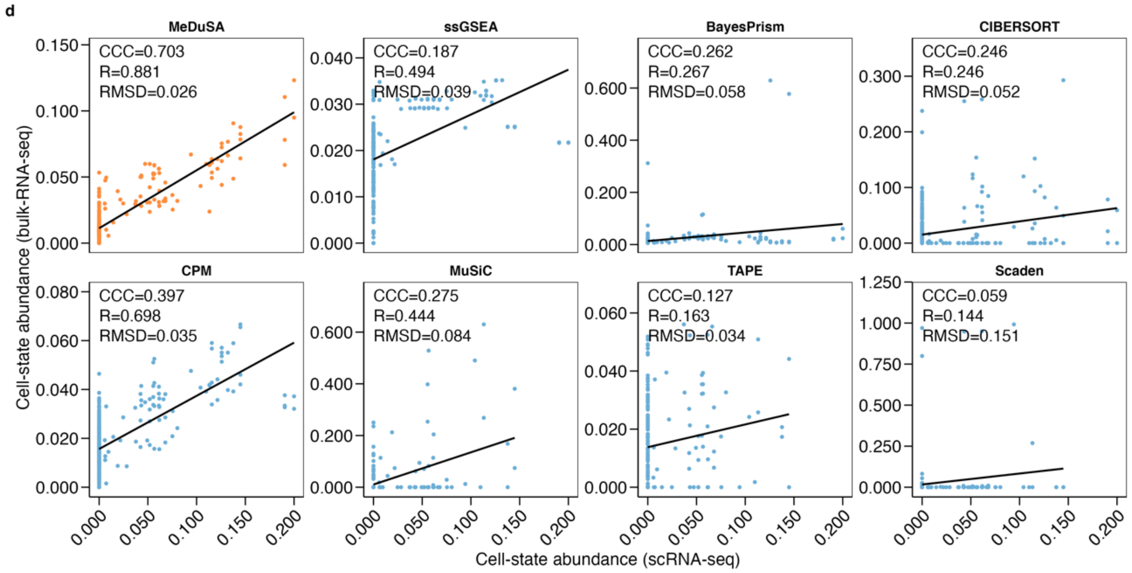

Supplementary Figure 12. Scatter plots comparing the cell-state abundance estimated from bulk RNA-seq data to that estimated from sample-matched scRNA-seq data. The results from different samples were pooled together into one scatter plot. The evaluation metrics (CCC, R, and RMSD) were computed using the pooled results. The x-axis represents cell-state abundance estimated from bulk RNA-seq data using the cellular deconvolution method shown on the top of each plot, and the y-axis represents cell-state abundance estimated from scRNA-seq data. Panels (a-d) show the results from the (a) human esophagus, (b) human bone marrow, (c) iPSC (c), and (d) hPSC data, respectively.

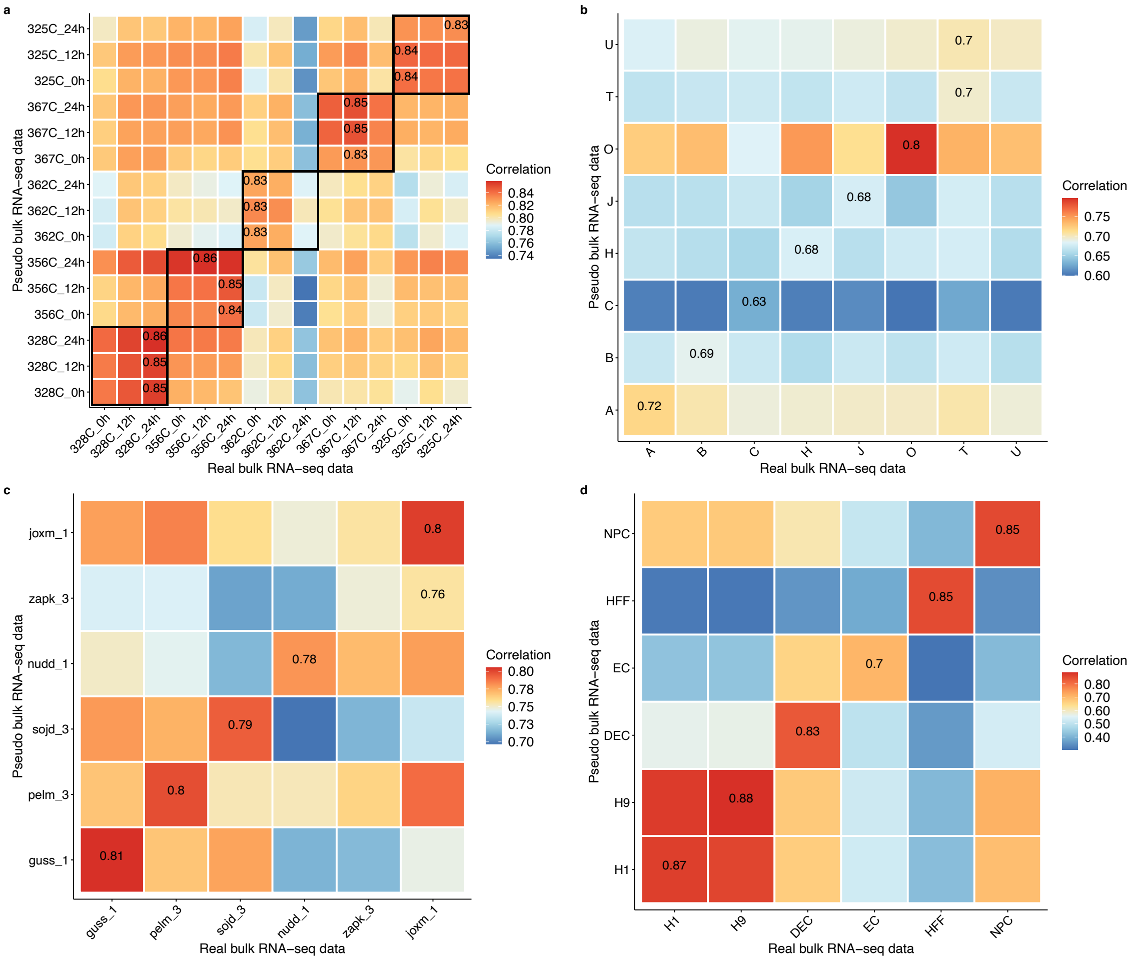

Supplementary Figure 13. Correlation of gene expression levels between real and the pseudo RNA-seq data. The pseudo bulk RNA-seq data were generated by summing up gene counts from all cells of the source scRNA-seq data. The correlation was computed on the log(TPM+1) scale. Panels (a-d) show the correlation heatmap in the (a) human esophagus, (b) human bone marrow, (c) iPSC, and (d) hPSC data, respectively. In the human esophagus data, the pseudo bulk RNA-seq data did not show the strongest correlation with the paired bulk RNA-seq data regarding storage time but displayed the highest correlation with the donor-matched bulk data.

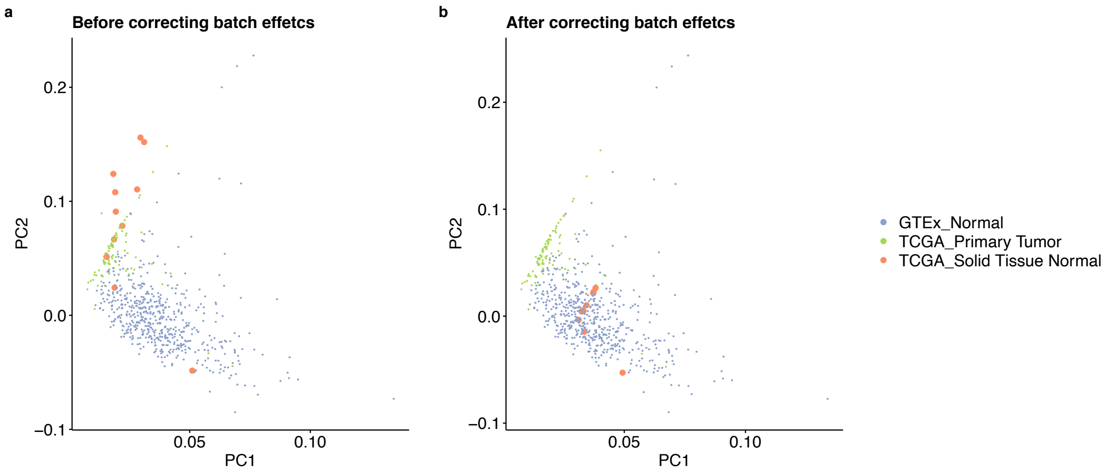

Supplementary Figure 14. PCA plot of TCGA and GTEx esophagus bulk RNA-seq data. Panels a and b show the PCA plot of the combined TCGA-GTEx esophagus bulk RNA-seq data ($n = 664$) before and after correcting batch effects, respectively. Each dot represents one sample, colored by the sample source. Batch effects between TCGA and GTEx datasets were corrected using ComBat-seq [49].

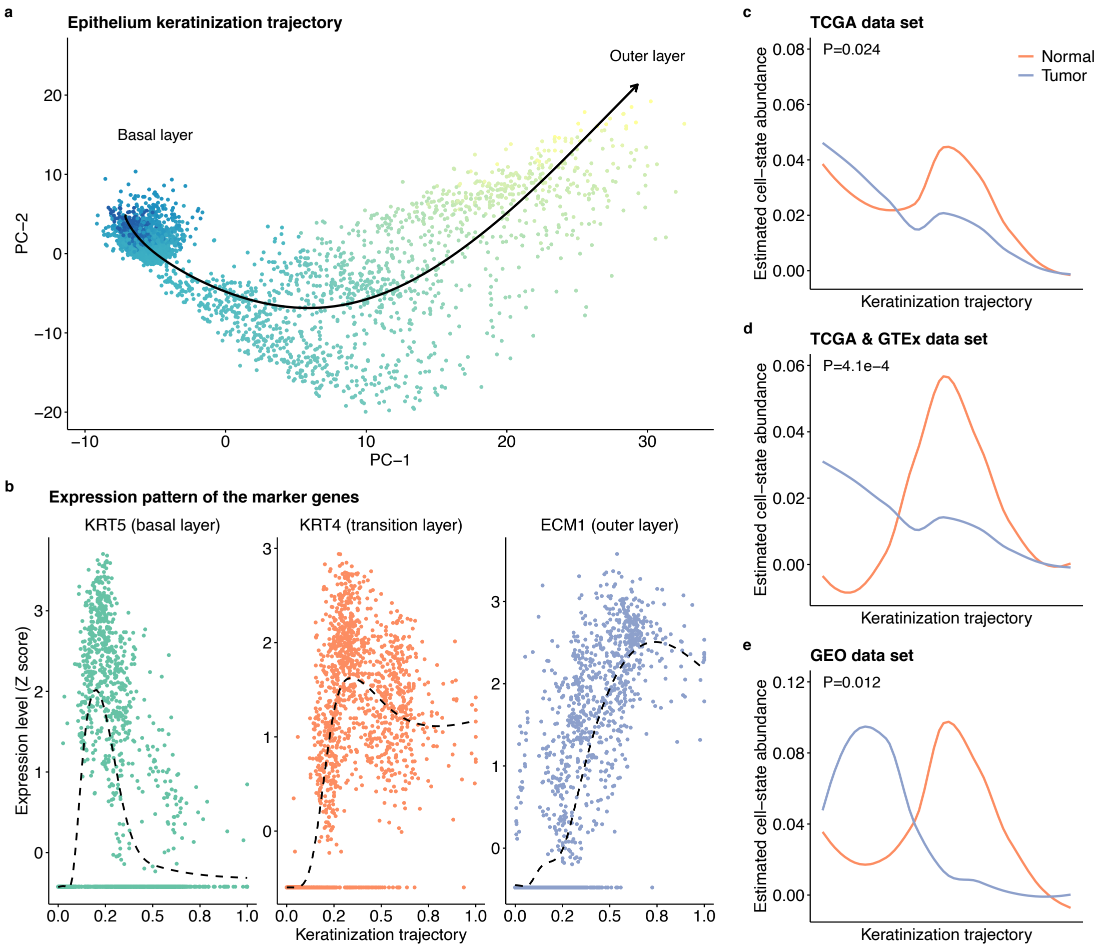

Supplementary Figure 15. Estimated epithelia abundance along the keratinization trajectory in normal and tumor esophagus tissues. (a) The keratinization trajectory of the epithelia in the reference scRNA-seq data (GSE173950). (b) The expression pattern of *KRT5* (marker gene of the basal layer), *KRT4* (marker gene of the transition layer), and *ECM1* (marker genes of the outer layer) confirmed the keratinization trajectory. (c-e) The cell-state abundance of epithelia estimated by MeDuSA using a dataset from TCGA (d, $n = 109$), a combined set of data from TCGA and GTEx (e, $n = 664$), and a dataset from the GEO (f, $n = 46$). The x-axis represents the keratinization trajectory, from the basal layer (left) to the outer layer (right). The curved line shows mean estimated cell-state abundance across individuals. The p-values were calculated using permutation-based MANOVA-Pro.

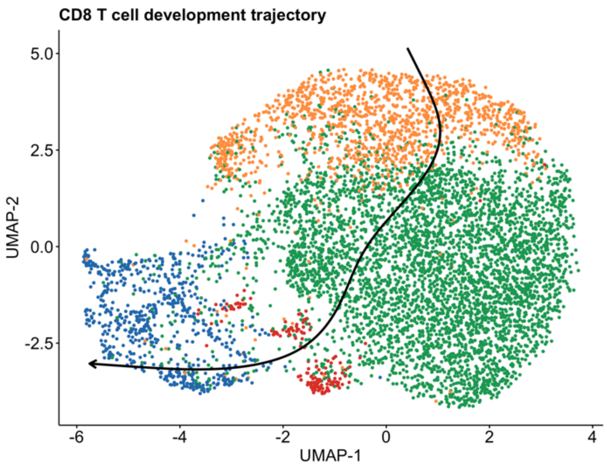

Supplementary Figure 16. The CD8+ T cells development trajectory annotated by slingshot. The black arrowed line represents the annotated cell-state trajectory. Colors represent subtypes of CD8+ T cells (orange, naive CD8+ T cells; green, effective memory CD8+ T cells; red, effective-exhaustion transition CD8+ T cells; blue, exhausted CD8+ T cells).

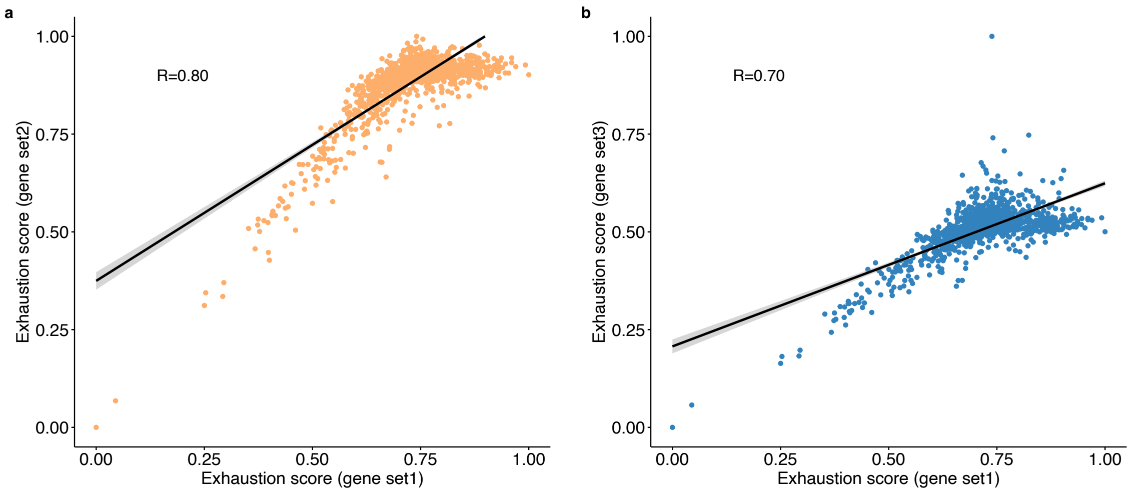

Supplementary Figure 17. CD8+ T cell exhaustion scores. Panels a and b show the correlation of the exhaustion score computed from gene set 1 with those from gene sets 2 and 3, respectively. For each CD8+ T cell, the exhaustion score was computed as the mean expression level of the exhaustion gene set minus that of the naïve gene set (**Methods**). Each dot represents one cell, the black line indicates the regression line, and the shaded area represents the 95% CI.

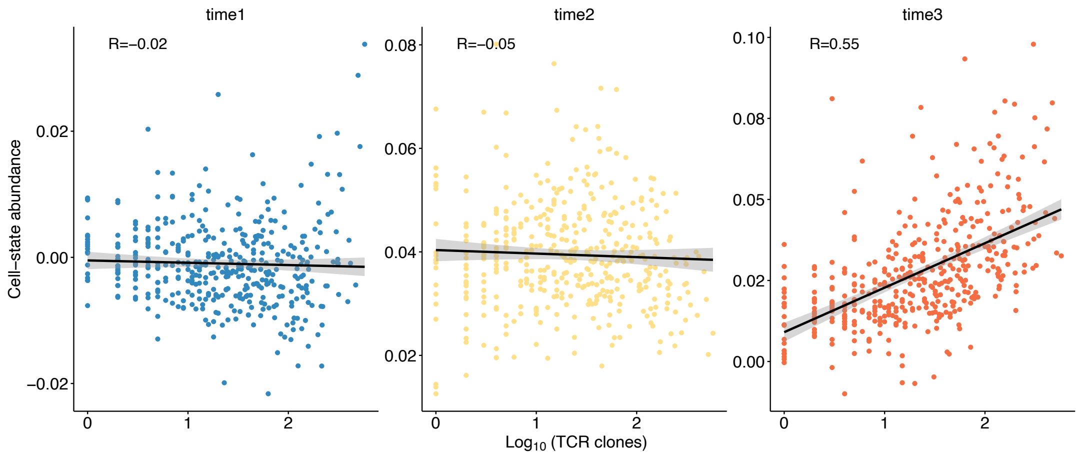

Supplementary Figure 18. Correlation between the TCR expansion level and the estimated cell-state abundance in each tertile of the exhaustion state. The x-axis represents the log transformed TCR expansion level. The y-axis shows the estimated cell-state abundance. The exhaustion-state tertiles are: time1 – the low-exhaustion state (0%-33% of the pseudotime), time2 – the medium-exhaustion state (33%-66% of the pseudotime), and time3 – the high-exhaustion state (66%-100% of the pseudotime). Each dot represents one cell, the black line indicates the regression line, and the shaded area represents the 95% CI.

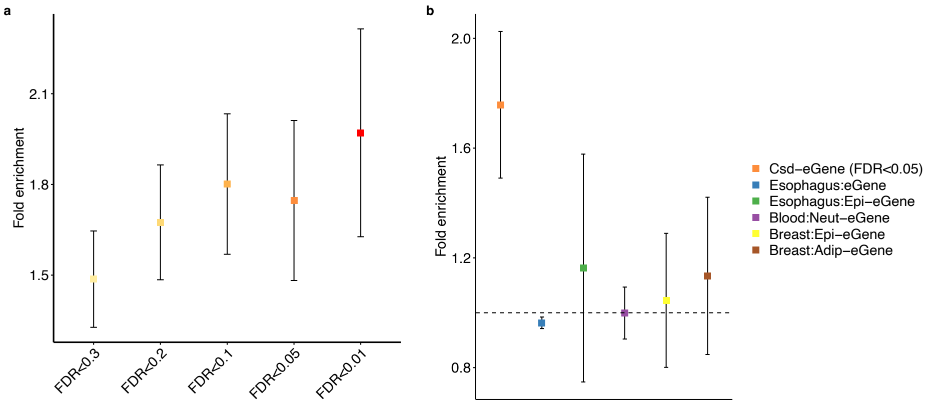

Supplementary Figure 19. Enrichment analysis of eGenes. The cell-state trajectory DEGs were identified using the GSE173950 scRNA-seq data. Based on this annotation, we replicated the eGene enrichment analysis described in the main text. (a) Enrichment of the csd-eGenes in the cell-state trajectory DEGs with different FDR thresholds. Each data point indicates the estimated fold enrichment, color-coded according to the corresponding FDR thresholds as displayed on the x-axis. The error bar represents the 95% CI computed using permutations (Methods: Enrichment of eGenes in DEGs). (b) Enrichment of the csd-eGenes (csd-eQTL FDR < 0.05), eGenes (obtained from the GTEx eQTL data with FDR < 0.05), or cell type-dependent eGenes (obtained from the GTEx cell type-dependent eQTL data with FDR < 0.05) in the cell-state trajectory DEGs. Each data point represents the estimated fold enrichment of eGenes, color-coded based on the corresponding tissue or cell type (Epi: epithelial cells, Neut: neutrophils, and Adip: adipose cells). Each error bar indicates the 95% CI for the estimated fold enrichment.

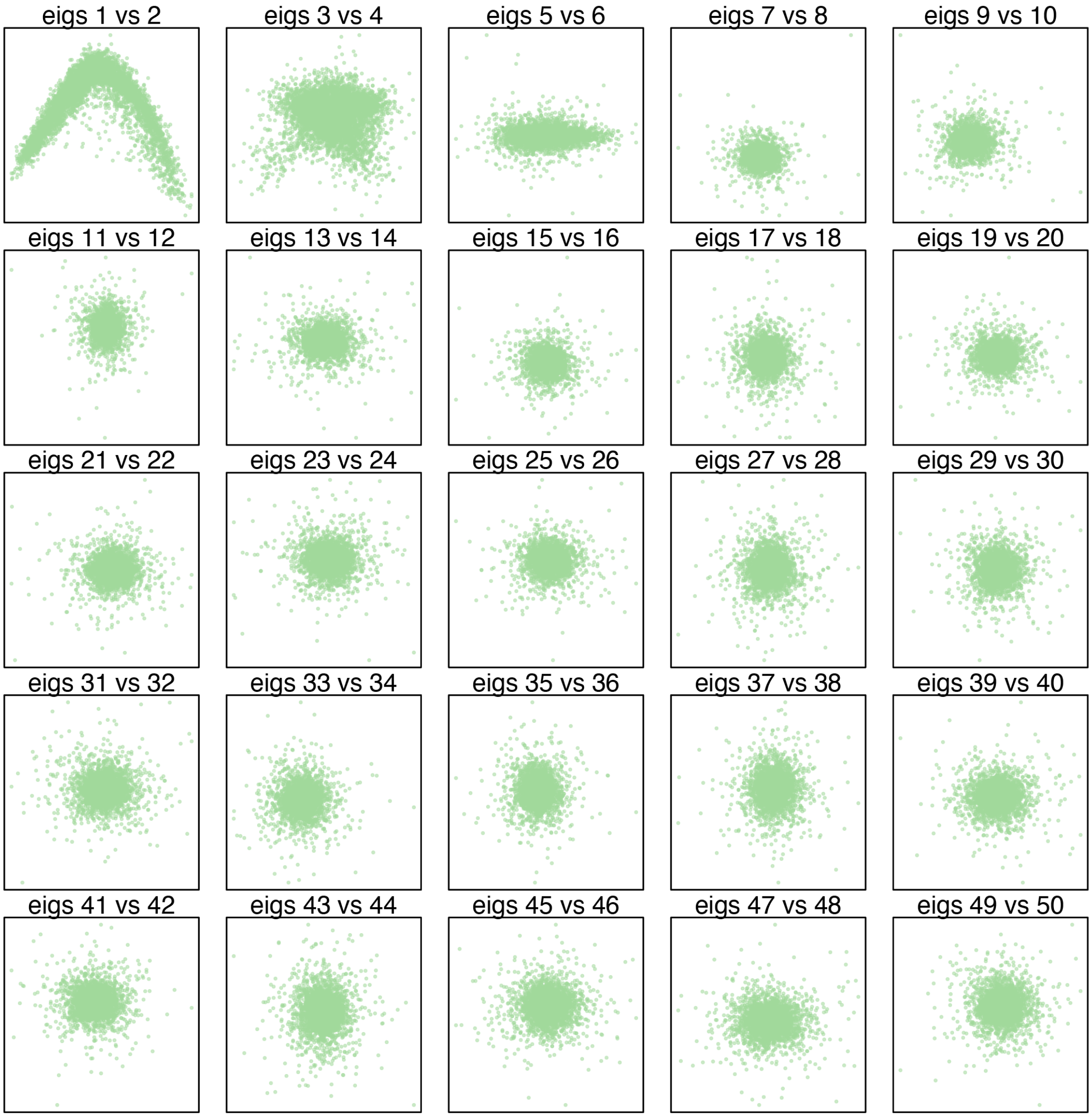

Supplementary Figure 20. PCA plots of the esophagus epithelia scATAC-seq data. The dimensional reduction analysis was performed using SnapATAC[43] (Methods). We used the top two eigenvectors for further analyses.

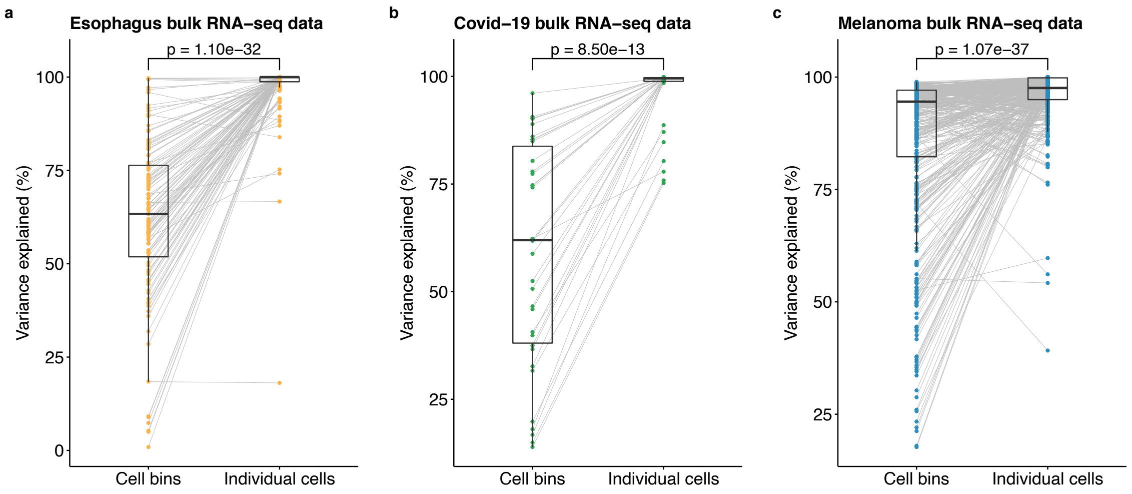

Supplementary Figure 21. Proportion of variance in bulk RNA-seq gene expression explained by fitting individual cells or the means of cell bins from the reference scRNA-seq data. Panels (a-c) show boxplots of the variance explained in the (a) esophagus ($n = 109$), (b) COVID-19 ($n = 34$), and (c) melanoma bulk RNA-seq data ($n = 430$), respectively. Each dot represents a sample in the bulk RNA-seq data, color-coded based on the corresponding tissue. The box indicates the IQR, the line within the box represents the median value, and the whiskers extend to data points within 1.5 times the IQR. The p-values were calculated using a two-sided Wilcoxon test.

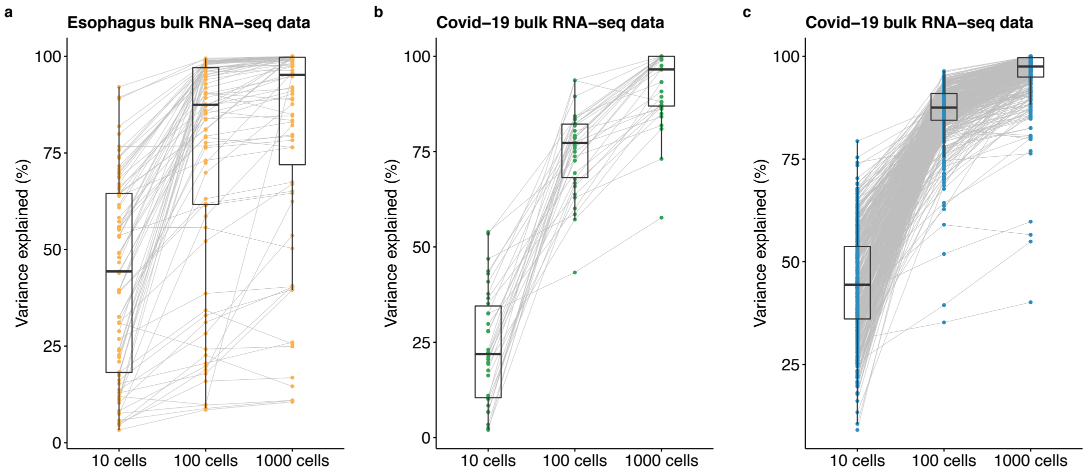

Supplementary Figure 22. Proportion of variance in bulk RNA-seq gene expression explained by fitting different number of individual cells from the reference scRNA-seq data. We grouped cells of the focal cell types into ten uniformly distributed cell bins over the cell-state trajectory and randomly sampled a subset of cells from each cell bin to be fitted in the random-effect component. Panels (a-c) show boxplots of the variance explained in the (a) esophagus (n=109), (b) COVID-19 (n=34), and (c) melanoma bulk RNA-seq data (n=430), respectively. Each dot represents a sample in the bulk RNA-seq data. The box indicates the IQR, the line within the box represents the median value, and the whiskers extend to data points within 1.5 times the IQR.

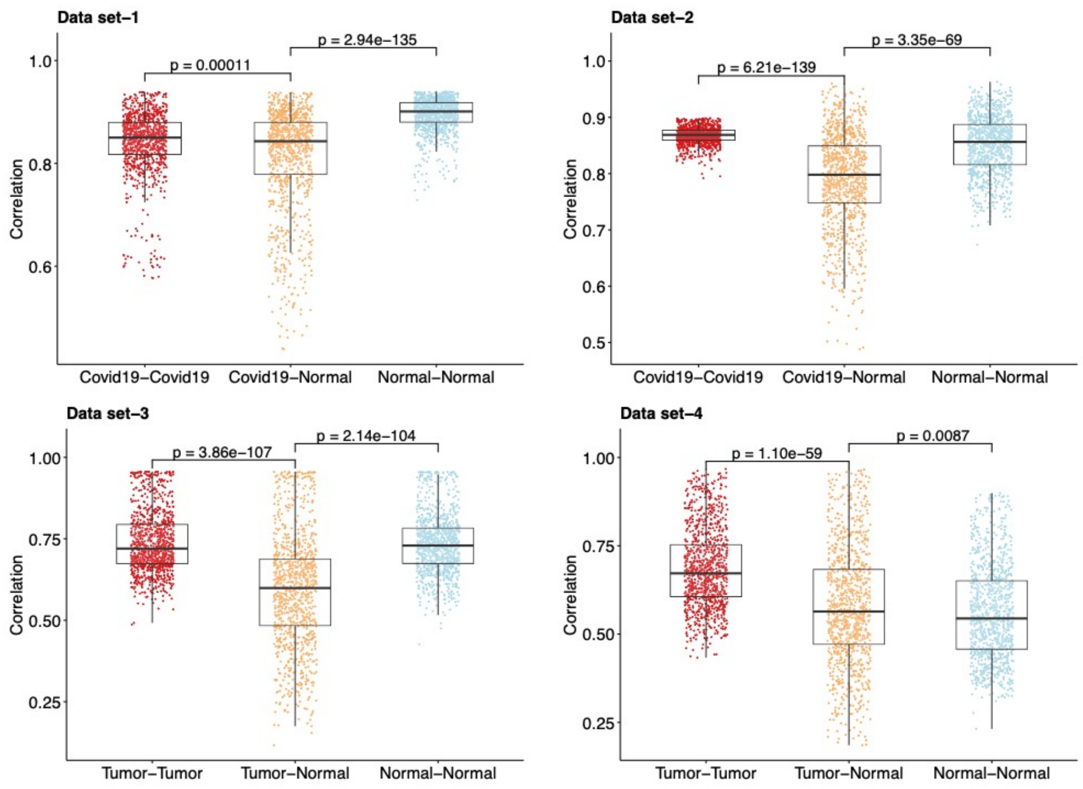

Supplementary Figure 23. With- and between-group correlation of gene expression in bulk RNA-seq data. For each bulk RNA-seq dataset, we randomly sampled 1,000 genes and repeated the random sampling 1,000 times. Each dot represents the mean correlation of gene expression across individuals within or between groups for the 1,000 genes. The box indicates the IQR, the line within the box represents the median value, and the whiskers extend to data points within 1.5 times the IQR. The p-values were calculated using a two-sided Wilcoxon test.

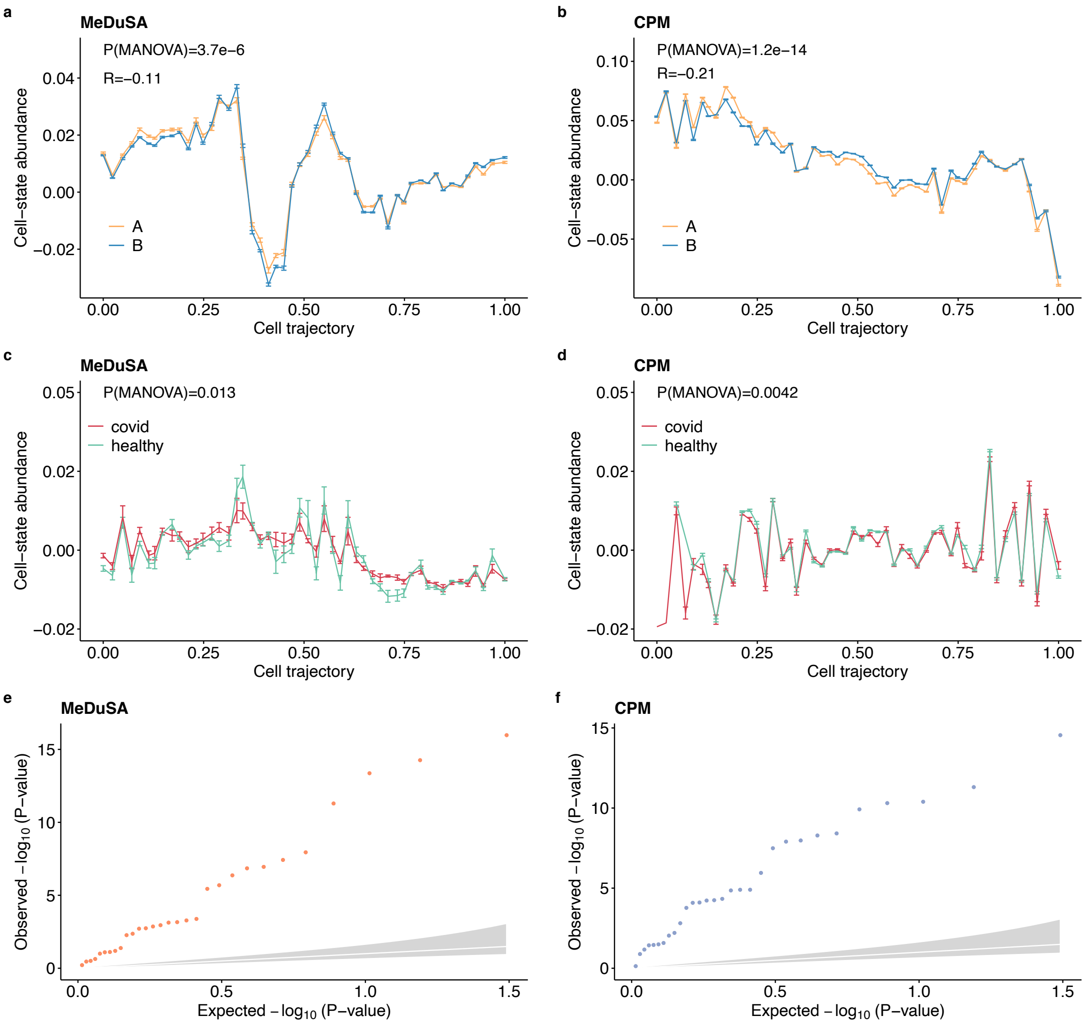

Supplementary Figure 24. Inflation in test statistics for association between the estimated cell-state abundance and case-control status when the reference RNA-seq data does not match the bulk RNA-seq data. (a-b) Test for differences in cell-state abundance between groups in simulations using a mismatched reference scRNA-seq data. We simulated the bulk RNA-seq data using the iPSC scRNA-seq data but performed cell-state deconvolution analysis using the esophagus scRNA-seq data as the reference. Although the estimated cell-state abundances are random (MeDuSA: R=-0.11, CPM: R=-0.21), the association test was significant for both MeDuSA and CPM. Each point represents the mean of estimated cell-state abundances across five simulation replicates, with the error bar indicating the standard deviation (SD). (c-d) Test for differences in cell-state abundance in real bulk RNA-seq (COVID-19) data using randomly generated reference scRNA-seq data and cell-state trajectory. Each point represents the mean estimated cell-state abundances across donors (n=34), with the error bar indicating the SD. (e-f) Q-Q Plot of p-values in real bulk RNA-seq data using

randomly generated reference scRNA-seq data and cell-state trajectory. The center white lines represent the expected p-values, and the grey shaded area represents the 95% CI. The p-values in all the above panels were calculated using a one-way MANOVA.

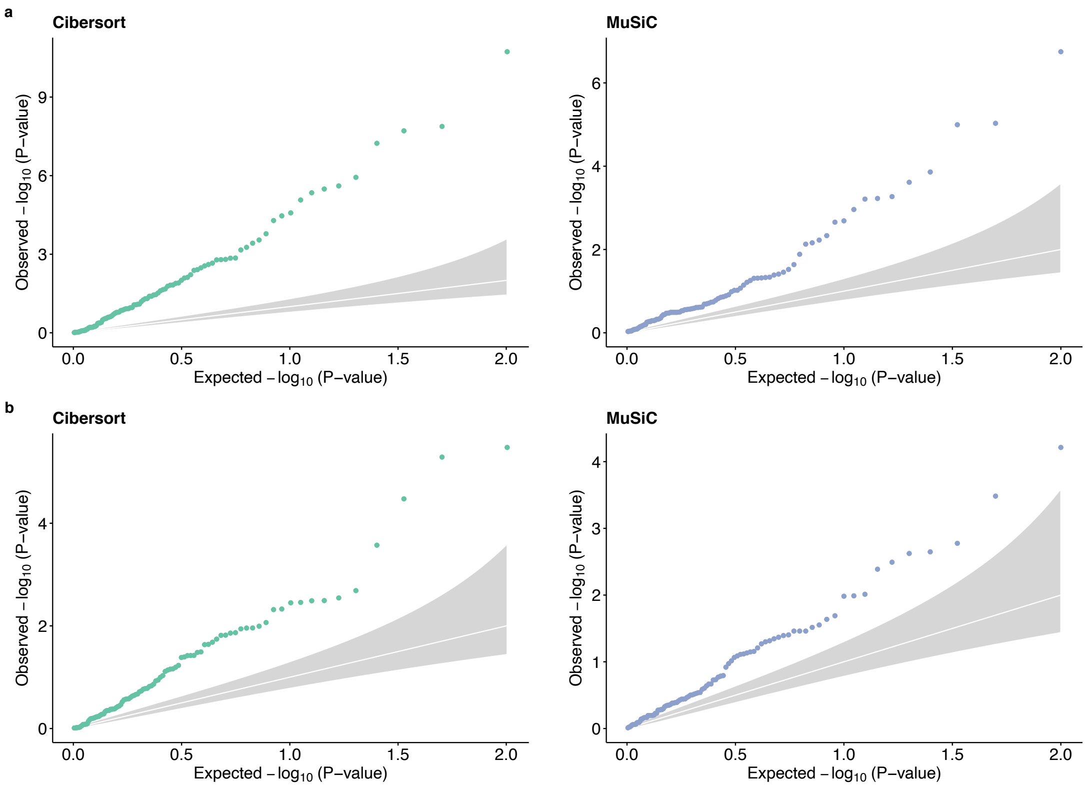

Supplementary Figure 25. Q-Q Plot of p-values for association between case-control status and estimated cell-type abundance (based on a random reference) in real bulk RNA-seq data. Both the reference scRNA-seq data and cell type labels were randomly generated with 100 repeats. Panels a and b show the Q-Q plot for real bulk RNA-seq data of COVID-19 (normal vs. COVID-19) and esophagus cancers (normal vs. tumor), respectively. The center white lines represent the expected p-values, and the grey shaded area represents the 95% CI. The p-value was computed using a two-sided t-test.

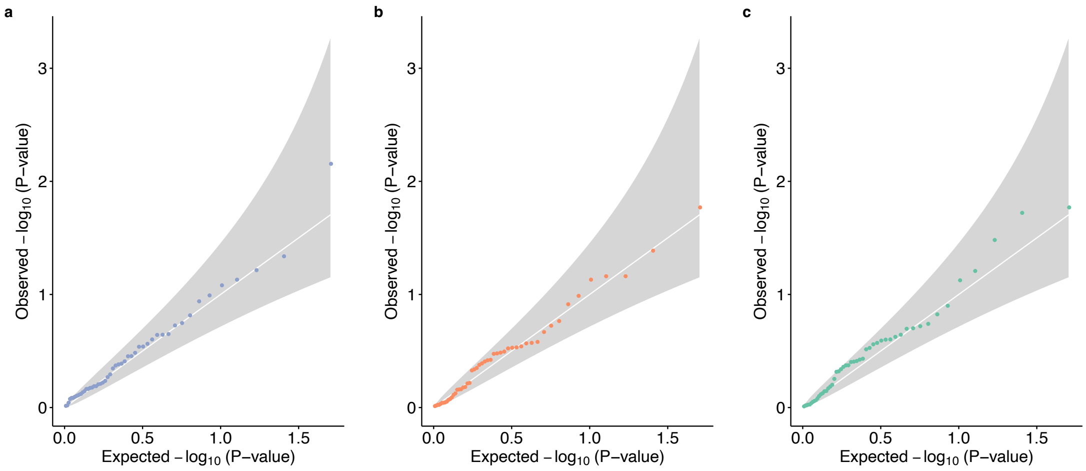

Supplementary Figure 26. Permutation p-values in simulations. (a) Q-Q plot of permutation p-values in control-control simulations when the reference scRNA-seq data match the simulated bulk RNA-seq data. (b) Q-Q plot of permutation p-values in control-control simulations when the reference scRNA-seq data and cell-state trajectory are randomly generated. (c) Q-Q plot of permutation p-values in case-control simulations when the reference scRNA-seq data and cell-state trajectory are randomly generated. The center white lines represent the expected p-values, and the grey shaded area represents the 95% CI. The p-value was computed using permutation-based MANOVA-Pro method (Methods: Testing for differences in cell-state abundances among groups; Correcting for inflation in association test).

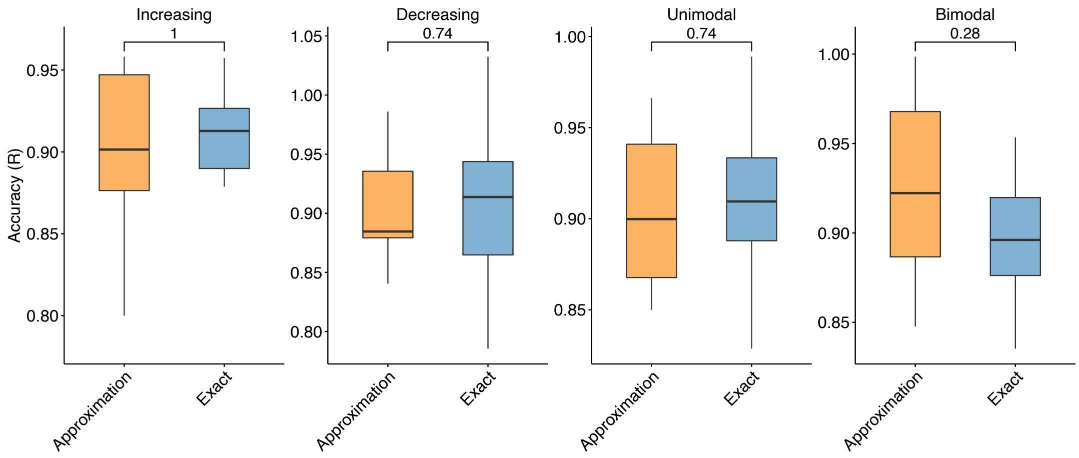

Supplementary Figure 27. Comparison of two implementations of MeDuSA based on the approximate and exact variance estimation approaches. The analysis was performed using the esophagus scRNA-seq data and the same simulation strategy described in the main text. Each boxplot represents deconvolution accuracy across 5 replicates. The approximate approach means that the variance components are estimated based on the null model that only includes the fixed-effect covariates and the random-effect component of all individual cells of the focal cell type. The exact approach means that variance components are estimated based on a full model that includes the fixed effect for a focal cell-state, the fixed-effect covariates, and the random-effect component of remaining individual cells of the focal cell type. The y-axis represents deconvolution accuracy (R). The text is the p-value of the mean difference in R between the approximate and the exact approaches. The box indicates the IQR, the line within the box represents the median value, and the whiskers extend to data points within 1.5 times the IQR. The p-values were calculated using a two-sided Wilcoxon test.

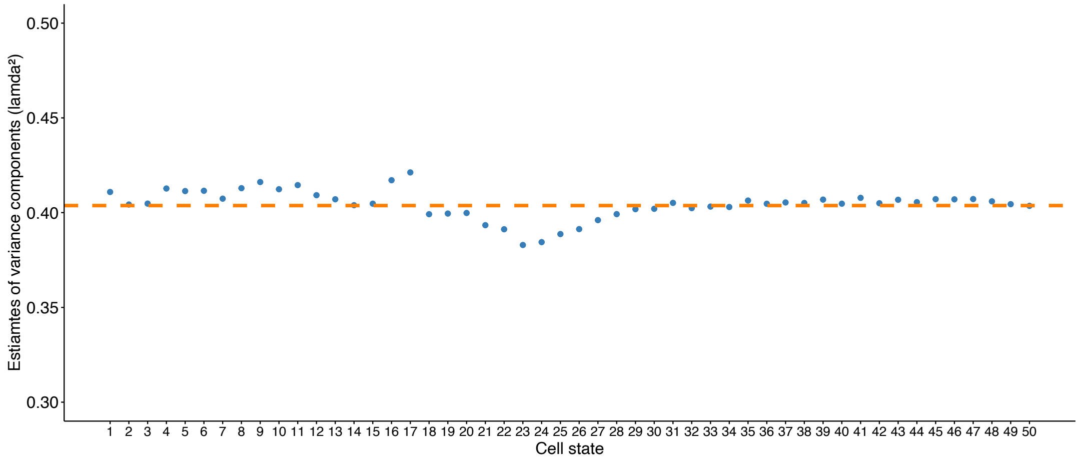

Supplementary Figure 28. Comparison of estimates of variance component from the approximate and exact approaches. The analysis was performed using the esophagus scRNA-seq data. Each blue point shows the estimate of variance component using the exact approach. The orange dashed line represents the estimate of variance components using the approximate approach. The x-axis represents the estimate of variance component when a cell state bin is fitted as a fixed effect.

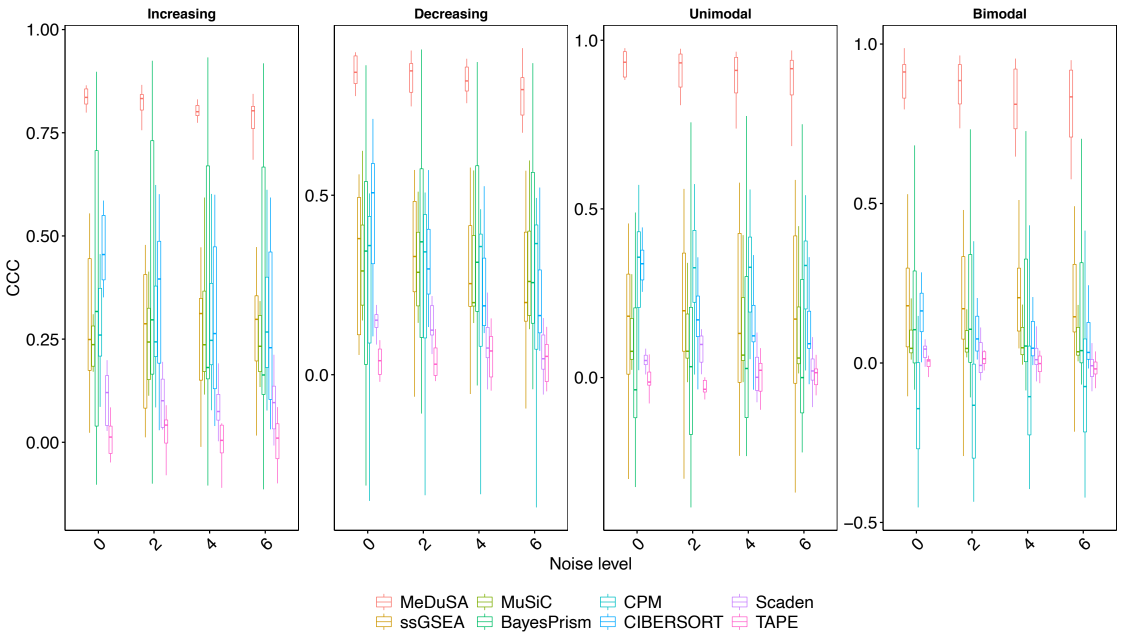

Supplementary Figure 29. Deconvolution accuracy of MeDuSA and other methods with log-normally distributed noises. To mimic differences in batch effects between scRNA-seq data and bulk RNA-seq data, we added log-normally distributed noises to the pseudo bulk RNA-seq data. To avoid unrealistic gene expression values, we removed extreme noises that are greater than 5-fold of the median[50]. We used 17 different scRNA-seq datasets as the simulation source data, and each simulation was replicated 5 times. The box indicates the interquartile range (IQR), the line within the box represents the median value, and the whiskers extend to data points within 1.5 times the IQR.

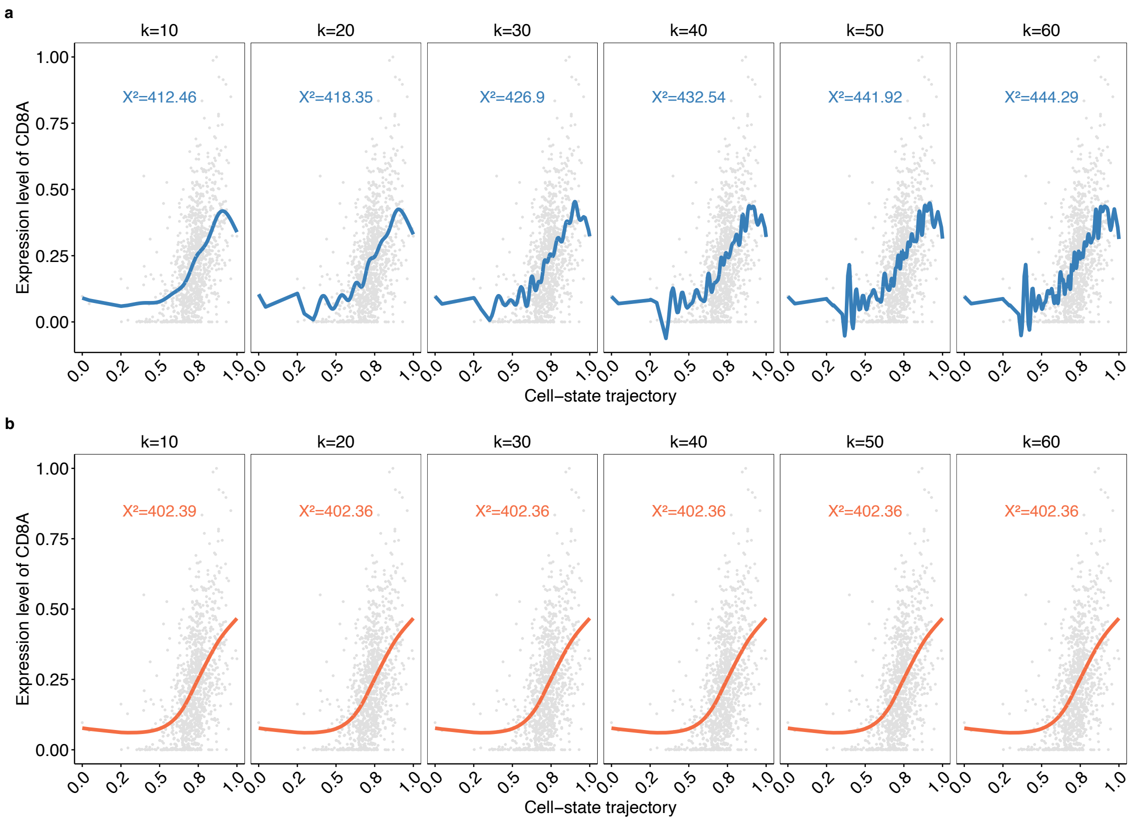

Supplementary Figure 30. Association of cellular-level expression of *CD8A* with cell-state trajectory. (a-b) We associated the cellular-level expression of *CD8A* with the exhaustion trajectory (estimated from the melanoma scRNA-seq data) using the GAM model based on the smoothing (panel a) or penalty (panel b) spline, with varying number of knots. The x-axis shows the exhaustion trajectory, from the naïve state (left) to the exhausted state (right). The y-axis shows the cellular-level expression of *CD8A* in the melanoma scRNA-seq data. The colored line represents the fitted association curve. The text shows the strength of the association (χ^2).

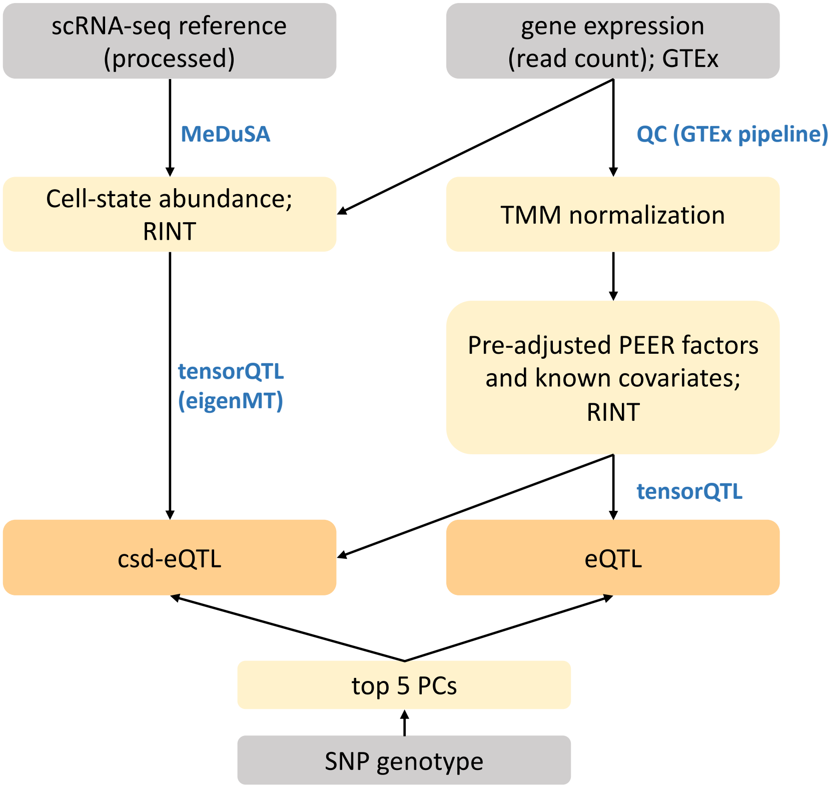

Supplementary Figure 31. The csd-eQTL analysis workflow. The eigenMT[51] and csd-eQTL test were performed using tensorQTL[52]. The quality control (QC) was performed following the GTEx eQTL mapping pipeline. RINT: rank-based inverse normal transformation.

Supplementary Table 1. The scRNA-seq and scATAC-seq data sets used in this study.

Accession	Platform	Species	Tissue	Cell number	Usage
GSE109774[53]	10x	Mouse	Bone marrow	3427	Simulation
CytoTRACE[25]	10x	C. elegans	Brain: neuron	10775	Simulation
CytoTRACE[25]	10x	C. elegans	Stem cell	12254	Simulation
GSE95753[54]	10x	Mouse	Dentate gyrus	24185	Simulation
CytoTRACE [25]	10x	C. elegans	Mesoderm	22370	Simulation
ERP016000[31]	10x	Human	iPSC	29494	Simulation/Application
GSE149938[55]	smart-seq2	Human	Bone marrow	7643	Simulation
GSE86146[56]	smart-seq2	Human	Embryo	1841	Simulation
CytoTRACE[25]	smart-seq2	Mouse	Whole intestine	1522	Simulation
GSE109774[53]	smart-seq2	Mouse	Bone marrow	4442	Simulation
GSE97391[57]	inDrop	Mouse	Neuron (direct)	2366	Simulation
GSE97391[57]	inDrop	Mouse	Neuron (standard)	2411	Simulation
GSE107122[58]	Drop-seq	Mouse	Neuron	5998	Simulation
GSE107910[59]	Drop-seq	Mouse	Thymus	10708	Simulation
CytoTRACE [25]	Drop-seq	Zebrafish	Embryo	39505	Simulation
GSE75330[60]	C1	Mouse	Oligodendrocyte	5053	Simulation
GSE75748[33]	C1	Human	Embryo	1018	Simulation
PRJEB31843[61]	10x	Human	Esophagus	58925	Simulation/Application
GSE120221[28]	10x	Human	Bone marrow	84881	Application
GSE75748[33]	Fluidigm C1	Human	hPSC	1018	Application
GSE173950	Drop-Seq	Human	Esophagus	37750	Application
GSE150728[62]	Seq-Well	Human	PBMC	44721	Application
GSE77940[63]	Smart-seq2	Human	Melanoma	2476	Application
GSE184462[42]	Zhang et.al.	Human	Esophagus	29121	Enrichment analysis

Supplementary Table 2. The bulk RNA-seq data sets used in this study.

Accession	Platform	Tissue/cell lines	Sample size
PRJEB31843[61]	Illumina HiSeq4000	Esophagus	15
GSE120444[28]	Illumina HiSeq 3000	Bone marrow	8
Cuomo et al.[32]	Illumina HiSeq2000	iPSC	6
GSE75748[33]	Illumina HiSeq 2500	hPSC	6
GTEx	Illumina HiSeq2000	Esophagus	555
TCGA	Illumina HiSeq2000	Esophagus	109
GSE130078[64]	Illumina HiSeq4000	Esophagus	46
GSE152418[65]	Illumina HiSeq2000	PBMC (COVID-19)	34
GSE157103[66]	Illumina HiSeq2500	PBMC (COVID-19)	100
GSE171110[67]	Illumina HiSeq 2500	PBMC (COVID-19)	54
GSE161777[68]	Illumina NovaSeq 6000	PBMC (COVID-19)	27
TCGA	Illumina HiSeq2000	Melanoma	430
Liu et al.[69]	Illumina HiSeq2000	Melanoma	77
GTEx	Illumina HiSeq2000	Blood	929
GTEx	Illumina HiSeq2000	Heart	861
GTEx	Illumina HiSeq2000	Liver	226
GTEx	Illumina HiSeq2000	Colon	779
GTEx	Illumina HiSeq2000	Small intestine	187
GTEx	Illumina HiSeq2000	Spleen	241
GTEx	Illumina HiSeq2000	Pancreas	328
GTEx	Illumina HiSeq2000	Kidney	89

Supplementary References

1. Trevor, H. & Robert, T. Generalized Additive Models. *Statistical Science* **1**, 297-310 (1986).
2. Wood, S.N. Stable and Efficient Multiple Smoothing Parameter Estimation for Generalized Additive Models. *Journal of the American Statistical Association* **99**, 673-686 (2004).
3. Golub, G.H., Heath, M. & Wahba, G. Generalized Cross-Validation as a Method for Choosing a Good Ridge Parameter. *Technometrics* **21**, 215-223 (1979).
4. Besag, J. Spatial Interaction and the Statistical Analysis of Lattice Systems. *Journal of the Royal Statistical Society. Series B (Methodological)* **36**, 192-236 (1974).
5. Brook, D. On the Distinction Between the Conditional Probability and the Joint Probability Approaches in the Specification of Nearest-Neighbour Systems. *Biometrika* **51**, 481-483 (1964).
6. Gilmour, A.R., Thompson, R. & Cullis, B.R. Average information REML: An efficient algorithm for variance parameter estimation in linear mixed models. *Biometrics* **51**, 1440-1450 (1995).
7. Wilk, A.J. *et al.* A single-cell atlas of the peripheral immune response in patients with severe COVID-19. *Nature Medicine* **26**, 1070-1076 (2020).
8. Wu, Y.L. *et al.* γδ T cells and their potential for immunotherapy. *Int J Biol Sci* **10**, 119-35 (2014).
9. Wauters, E. *et al.* Discriminating mild from critical COVID-19 by innate and adaptive immune single-cell profiling of bronchoalveolar lavages. *Cell Res* **31**, 272-290 (2021).
10. Kusnadi, A. *et al.* Severely ill patients with COVID-19 display impaired exhaustion features in SARS-CoV-2–reactive CD8+ T cells. *Science Immunology* **6**, eabe4782 (2021).
11. Zhang, J.-Y. *et al.* Single-cell landscape of immunological responses in patients with COVID-19. *Nature Immunology* **21**, 1107-1118 (2020).
12. Rha, M.-S. & Shin, E.-C. Activation or exhaustion of CD8+ T cells in patients with COVID-19. *Cellular & Molecular Immunology* **18**, 2325-2333 (2021).
13. Arunachalam, P.S. *et al.* Systems biological assessment of immunity to mild versus severe COVID-19 infection in humans. *Science* **369**, 1210 (2020).
14. Wauters, E. *et al.* Discriminating mild from critical COVID-19 by innate and adaptive immune single-cell profiling of bronchoalveolar lavages. *Cell Research* **31**, 272-290 (2021).
15. Chen, Z. & John Wherry, E. T cell responses in patients with COVID-19. *Nature Reviews Immunology* **20**, 529-536 (2020).
16. Kusnadi, A. *et al.* Severely ill patients with COVID-19 display impaired exhaustion features in SARS-CoV-2–reactive CD8⁺ T cells. *Science Immunology* **6**, eabe4782 (2021).
17. Lévy, Y. *et al.* CD177, a specific marker of neutrophil activation, is associated with coronavirus disease 2019 severity and death. *iScience* **24**, 102711-102711 (2021).

18. Overmyer, K.A. *et al.* Large-Scale Multi-omic Analysis of COVID-19 Severity. *Cell systems* **12**, 23-40.e7 (2021).
19. Bernardes, J.P. *et al.* Longitudinal Multi-omics Analyses Identify Responses of Megakaryocytes, Erythroid Cells, and Plasmablasts as Hallmarks of Severe COVID-19. *Immunity* **53**, 1296-1314.e9 (2020).
20. Chen, Y. *et al.* Deep autoencoder for interpretable tissue-adaptive deconvolution and cell-type-specific gene analysis. *Nature Communications* **13**, 6735 (2022).
21. Stuart, T. *et al.* Comprehensive Integration of Single-Cell Data. *Cell* **177**, 1888-1902.e21 (2019).
22. Mabbott, N.A., Baillie, J.K., Brown, H., Freeman, T.C. & Hume, D.A. An expression atlas of human primary cells: inference of gene function from coexpression networks. *BMC Genomics* **14**, 632 (2013).
23. Aran, D. *et al.* Reference-based analysis of lung single-cell sequencing reveals a transitional profibrotic macrophage. *Nature Immunology* **20**, 163-172 (2019).
24. Street, K. *et al.* Slingshot: cell lineage and pseudotime inference for single-cell transcriptomics. *BMC Genomics* **19**, 477 (2018).
25. Gulati, G.S. *et al.* Single-cell transcriptional diversity is a hallmark of developmental potential. *Science* **367**, 405-411 (2020).
26. Bergen, V., Lange, M., Peidli, S., Wolf, F.A. & Theis, F.J. Generalizing RNA velocity to transient cell states through dynamical modeling. *Nature Biotechnology* **38**, 1408-1414 (2020).
27. Madissoon, E. *et al.* scRNA-seq assessment of the human lung, spleen, and esophagus tissue stability after cold preservation. *Genome Biology* **21**, 1 (2019).
28. Oetjen, K.A. *et al.* Human bone marrow assessment by single-cell RNA sequencing, mass cytometry, and flow cytometry. *JCI Insight* **3**(2018).
29. McGinnis, C.S., Murrow, L.M. & Gartner, Z.J. DoubletFinder: Doublet Detection in Single-Cell RNA Sequencing Data Using Artificial Nearest Neighbors. *Cell Systems* **8**, 329-337.e4 (2019).
30. Lappalainen, T. *et al.* Transcriptome and genome sequencing uncovers functional variation in humans. *Nature* **501**, 506-511 (2013).
31. Cuomo, A.S.E. *et al.* Single-cell RNA-sequencing of differentiating iPS cells reveals dynamic genetic effects on gene expression. *Nature Communications* **11**, 810 (2020).
32. Cuomo, A.S.E. *et al.* Optimizing expression quantitative trait locus mapping workflows for single-cell studies. *Genome Biology* **22**, 188 (2021).
33. Chu, L.F. *et al.* Single-cell RNA-seq reveals novel regulators of human embryonic stem cell differentiation to definitive endoderm. *Genome Biol* **17**, 173 (2016).
34. Zheng, L. *et al.* Pan-cancer single-cell landscape of tumor-infiltrating T cells. *Science* **374**, abe6474 (2021).
35. van den Brink, S.C. *et al.* Single-cell sequencing reveals dissociation-induced gene expression in tissue subpopulations. *Nature Methods* **14**, 935-936 (2017).
36. Tirosh, I. *et al.* Dissecting the multicellular ecosystem of metastatic melanoma by

single-cell RNA-seq. *Science (New York, N.Y.)* **352**, 189-196 (2016).
37. Racle, J., de Jonge, K., Baumgaertner, P., Speiser, D.E. & Gfeller, D. Simultaneous enumeration of cancer and immune cell types from bulk tumor gene expression data. *eLife* **6**, e26476 (2017).
38. Chen, S., Zhou, Y., Chen, Y. & Gu, J. fastp: an ultra-fast all-in-one FASTQ preprocessor. *Bioinformatics* **34**, i884-i890 (2018).
39. Dobin, A. *et al.* STAR: ultrafast universal RNA-seq aligner. *Bioinformatics (Oxford, England)* **29**, 15-21 (2013).
40. Li, B. & Dewey, C.N. RSEM: accurate transcript quantification from RNA-Seq data with or without a reference genome. *BMC Bioinformatics* **12**, 323 (2011).
41. Bolotin, D.A. *et al.* MiXCR: software for comprehensive adaptive immunity profiling. *Nature Methods* **12**, 380-381 (2015).
42. Zhang, K. *et al.* A single-cell atlas of chromatin accessibility in the human genome. *Cell* **184**, 5985-6001.e19 (2021).
43. Fang, R. *et al.* Comprehensive analysis of single cell ATAC-seq data with SnapATAC. *Nature Communications* **12**, 1337 (2021).
44. Amemiya, H.M., Kundaje, A. & Boyle, A.P. The ENCODE Blacklist: Identification of Problematic Regions of the Genome. *Scientific Reports* **9**, 9354 (2019).
45. Haghverdi, L., Buettner, F. & Theis, F.J. Diffusion maps for high-dimensional single-cell analysis of differentiation data. *Bioinformatics* **31**, 2989-98 (2015).
46. Korsunsky, I. *et al.* Fast, sensitive and accurate integration of single-cell data with Harmony. *Nature Methods* **16**, 1289-1296 (2019).
47. Traag, V.A., Waltman, L. & van Eck, N.J. From Louvain to Leiden: guaranteeing well-connected communities. *Scientific Reports* **9**, 5233 (2019).
48. Zhang, Y. *et al.* Model-based Analysis of ChIP-Seq (MACS). *Genome Biology* **9**, R137 (2008).
49. Zhang, Y., Parmigiani, G. & Johnson, W.E. ComBat-seq: batch effect adjustment for RNA-seq count data. *NAR Genomics and Bioinformatics* **2**, lqaa078 (2020).
50. Chu, T., Wang, Z., Pe'er, D. & Danko, C.G. Cell type and gene expression deconvolution with BayesPrism enables Bayesian integrative analysis across bulk and single-cell RNA sequencing in oncology. *Nature Cancer* **3**, 505-517 (2022).
51. Davis, J.R. *et al.* An Efficient Multiple-Testing Adjustment for eQTL Studies that Accounts for Linkage Disequilibrium between Variants. *Am J Hum Genet* **98**, 216-24 (2016).
52. Taylor-Weiner, A. *et al.* Scaling computational genomics to millions of individuals with GPUs. *Genome Biology* **20**, 228 (2019).
53. Single-cell transcriptomics of 20 mouse organs creates a Tabula Muris. *Nature* **562**, 367-372 (2018).
54. Hochgerner, H., Zeisel, A., Lönnerberg, P. & Linnarsson, S. Conserved properties of dentate gyrus neurogenesis across postnatal development revealed by single-cell RNA sequencing. *Nat Neurosci* **21**, 290-299 (2018).

55. Xie, X. *et al.* Single-cell transcriptomic landscape of human blood cells. *Natl Sci Rev* **8**, nwaa180 (2021).
56. Li, L. *et al.* Single-Cell RNA-Seq Analysis Maps Development of Human Germline Cells and Gonadal Niche Interactions. *Cell Stem Cell* **20**, 858-873.e4 (2017).
57. Briggs, J.A. *et al.* Mouse embryonic stem cells can differentiate via multiple paths to the same state. *Elife* **6**(2017).
58. Yuzwa, S.A. *et al.* Developmental Emergence of Adult Neural Stem Cells as Revealed by Single-Cell Transcriptional Profiling. *Cell Rep* **21**, 3970-3986 (2017).
59. Kernfeld, E.M. *et al.* A Single-Cell Transcriptomic Atlas of Thymus Organogenesis Resolves Cell Types and Developmental Maturation. *Immunity* **48**, 1258-1270.e6 (2018).
60. Marques, S. *et al.* Oligodendrocyte heterogeneity in the mouse juvenile and adult central nervous system. *Science* **352**, 1326-1329 (2016).
61. Denisenko, E. *et al.* Systematic assessment of tissue dissociation and storage biases in single-cell and single-nucleus RNA-seq workflows. *Genome Biol* **21**, 130 (2020).
62. Wilk, A.J. *et al.* A single-cell atlas of the peripheral immune response in patients with severe COVID-19. *Nat Med* **26**, 1070-1076 (2020).
63. Tirosh, I. *et al.* Dissecting the multicellular ecosystem of metastatic melanoma by single-cell RNA-seq. *Science* **352**, 189-96 (2016).
64. You, B.H. *et al.* HERES, a lncRNA that regulates canonical and noncanonical Wnt signaling pathways via interaction with EZH2. *Proc Natl Acad Sci U S A* **116**, 24620-24629 (2019).
65. Arunachalam, P.S. *et al.* Systems biological assessment of immunity to mild versus severe COVID-19 infection in humans. *Science* **369**, 1210-1220 (2020).
66. Overmyer, K.A. *et al.* Large-Scale Multi-omic Analysis of COVID-19 Severity. *Cell Syst* **12**, 23-40.e7 (2021).
67. Lévy, Y. *et al.* CD177, a specific marker of neutrophil activation, is associated with coronavirus disease 2019 severity and death. *iScience* **24**, 102711 (2021).
68. Bernardes, J.P. *et al.* Longitudinal Multi-omics Analyses Identify Responses of Megakaryocytes, Erythroid Cells, and Plasmablasts as Hallmarks of Severe COVID-19. *Immunity* **53**, 1296-1314.e9 (2020).
69. Liu, D. *et al.* Integrative molecular and clinical modeling of clinical outcomes to PD1 blockade in patients with metastatic melanoma. *Nat Med* **25**, 1916-1927 (2019).
