# Supplementary material for: Mixed model-based deconvolution of cell-state abundances (MeDuSA) along a one-dimensional trajectory
Source: Nat Comput Sci. 2023 Jul 13;3(7):630–43. doi: 10.1038/s43588-023-00487-2 (PMC10766563; doi:10.1038/s43588-023-00487-2)
Supplement: Supplementary file 2 — Reporting Summary [file 43588_2023_487_MOESM2_ESM.pdf]

nature portfolio

Corresponding author(s): Jian Yang

Last updated by author(s): Jun 4, 2023

Reporting Summary

Nature Portfolio wishes to improve the reproducibility of the work that we publish. This form provides structure for consistency and transparency in reporting. For further information on Nature Portfolio policies, see our Editorial Policies and the Editorial Policy Checklist.

Statistics

For all statistical analyses, confirm that the following items are present in the figure legend, table legend, main text, or Methods section.

n/a	Confirmed	
☐	☒	The exact sample size (*n*) for each experimental group/condition, given as a discrete number and unit of measurement
☐	☒	A statement on whether measurements were taken from distinct samples or whether the same sample was measured repeatedly
☐	☒	The statistical test(s) used AND whether they are one- or two-sided<br>*Only common tests should be described solely by name; describe more complex techniques in the Methods section.*
☐	☒	A description of all covariates tested
☐	☒	A description of any assumptions or corrections, such as tests of normality and adjustment for multiple comparisons
☐	☒	A full description of the statistical parameters including central tendency (e.g. means) or other basic estimates (e.g. regression coefficient) AND variation (e.g. standard deviation) or associated estimates of uncertainty (e.g. confidence intervals)
☐	☒	For null hypothesis testing, the test statistic (e.g. *F*, *t*, *r*) with confidence intervals, effect sizes, degrees of freedom and *P* value noted<br>*Give P values as exact values whenever suitable.*
☒	☐	For Bayesian analysis, information on the choice of priors and Markov chain Monte Carlo settings
☒	☐	For hierarchical and complex designs, identification of the appropriate level for tests and full reporting of outcomes
☐	☒	Estimates of effect sizes (e.g. Cohen's *d*, Pearson's *r*), indicating how they were calculated

Our web collection on statistics for biologists contains articles on many of the points above.

Software and code

Policy information about availability of computer code

Data collection	No data-collection software was used.
Data analysis	We implemented our method in a R package called MeDuSA. The source code is freely available at https://github.com/LeonSong1995/MeDuSA. CellRanger v6.1.2, R v4.1.0 (package: Seurat v3, SingleR v1.8.0, Slingshot v2.8.0, SCORPIUS v1.0.8, TSCAN v1.38.0, SnapATAC v2.0, CytoTrace v0.3.3), and Python v3.8 (package: scVelo v0.2.5) were used for the quality control and statistical analyses of the scRNA-seq and scATAC-seq data. MACS2 v2.2.7 was used for calling chromatin peaks of the scATAC-seq data. STAR v2.7.9 , RSEM v1.1.17 and Fastp v 0.23.2 were used for the quality control and statistical analyses of the bulk RNA-seq data. MIXCR v3.0.13 was used for the T cell receptor expansion analyses. R v4.1.0 (package: scBio v0.1.5, MuSiC v1.00, Cibersort v1.00, EPIC v1.16, GSVA v1.48.1, and BayesPrism v2.0) and Python v3.10 (package: Scaden v1.10 and TAPE v1.12) were used for the cell type (state) deconvolution analyses. Plink v2.0 was used for the quality control of the genotype data. TensorQTL v1.0.7 was used for the eQTL analysis.

For manuscripts utilizing custom algorithms or software that are central to the research but not yet described in published literature, software must be made available to editors and reviewers. We strongly encourage code deposition in a community repository (e.g. GitHub). See the Nature Portfolio guidelines for submitting code & software for further information.

Data

Policy information about availability of data

All manuscripts must include a data availability statement. This statement should provide the following information, where applicable:

- Accession codes, unique identifiers, or web links for publicly available datasets
- A description of any restrictions on data availability
- For clinical datasets or third party data, please ensure that the statement adheres to our policy

All the scRNA-seq, scATAC-seq, and bulk RNA-seq data used in this study are available in the public domain with the relevant information summarized in Supplementary Tables 1-2. The GTEx genotype data is available at: https://gtexportal.org/home/protectedDataAccess. The GTEx eQTLs summary data is available at: https://gtexportal.org/home/datasets. The csd-eQTLs summary data is available at zenodo: https://doi.org/10.5281/zenodo.8018006. The GRCh38 genome is available at: https://www.ncbi.nlm.nih.gov/projects/genome/guide/human. The GENECODE-v38 transcriptome reference is available at:https://www.gencodegenes.org/human. Source data for Figures 2-6 and Extended Data Figures 1-5 are available with this manuscript.

Human research participants

Policy information about studies involving human research participants and Sex and Gender in Research.

Reporting on sex and gender	We analyzed existing data sets and collected the age and gender from the corresponding public data domain. We have included age and gender as covariates in analysis.
Population characteristics	Our study involved publicly available datasets (e.g., GTEx, TCGA and existing summary statistics). We have included data from different ancestries.
Recruitment	We analyzed existing data sets. Thus, no recruitment was performed.
Ethics oversight	Ethics Committee of Westlake University (No. 20200722YJ001)

Note that full information on the approval of the study protocol must also be provided in the manuscript.

Field-specific reporting

Please select the one below that is the best fit for your research. If you are not sure, read the appropriate sections before making your selection.

☒ Life sciences ☐ Behavioural & social sciences ☐ Ecological, evolutionary & environmental sciences

For a reference copy of the document with all sections, see nature.com/documents/nr-reporting-summary-flat.pdf

Life sciences study design

All studies must disclose on these points even when the disclosure is negative.

Sample size	This study uses twenty-four scRNA-seq and twenty-one bulk RNA-seq datasets throughout the simulation, validation, and four applications. The sample sizes of all the datasets have been described in Supplementary Tales 1 and 2. In the simulation study, the cell number across seventeen scRNA-seq datasets totaled 185,012, determined by the maximum number of eligible cells after implementing quality control measures. For the validation study, we included 4,684 samples from twelve distinct tissues (cell lines), which were determined by the maximum number of eligible samples in the respective datasets. For the case studies, the sample size amounted to 711 for the esophagus data, 215 for the COVID-19 data, and 507 for the melanoma data, based on the maximum number of eligible samples in the corresponding datasets. For the csd-eQTL mapping, the sample size is 497, which was determined by the maximum number of unrelated individuals possessing both SNP genotype and esophagus bulk RNA-seq data in the GTEx dataset.
Data exclusions	For the scRNA-seq data, we excluded cells with >4500 and <2000 expressed genes (potential duplets or empty droplets). For the bulk RNA-seq data, we excluded individuals with ambiguous clinical diagnoses.
Replication	We repeated the simulation with each specific setting multiple times to assess the robustness of the deconvolution method. All the simulation replications were successfully performed. In the real data application for esophageal samples, we reproduced the results in three distinct bulk RNA-seq datasets and two scRNA-seq datasets. Regarding the real data application for COVID-19, we replicated the findings across three bulk RNA-seq datasets. For csd-eQTL mapping, we repeated the enrichment analysis using two distinct scRNA-seq datasets. The results demonstrated consistency across all replications.
Randomization	We performed analyses of the existing datasets; therefore, no randomization was implemented with regard to data generation. In the simulation analysis, we divided the scRNA-seq dataset into two sections at random. One section was then randomly designated as the simulation source data, and the other portion used as the reference data for deconvolution analysis.
Blinding	We performed analyses of the existing datasets, and as such,no blinding measures were implemented in this study.

Reporting for specific materials, systems and methods

We require information from authors about some types of materials, experimental systems and methods used in many studies. Here, indicate whether each material, system or method listed is relevant to your study. If you are not sure if a list item applies to your research, read the appropriate section before selecting a response.

Materials & experimental systems

n/a	Involved in the study
☒	☐ Antibodies
☒	☐ Eukaryotic cell lines
☒	☐ Palaeontology and archaeology
☒	☐ Animals and other organisms
☒	☐ Clinical data
☒	☐ Dual use research of concern

Methods

n/a	Involved in the study
☒	☐ ChIP-seq
☒	☐ Flow cytometry
☒	☐ MRI-based neuroimaging
